# Drug Safety and Polypharmacy Signals in Ibrutinib-Treated Chronic Lymphocytic Leukemia/Small Lymphocytic Lymphoma: Age- and Sex-Specific FAERS Analysis of CYP3A Modifier and Antithrombotic Co-Exposure

**DOI:** 10.3390/jcm15166370

**Published:** 2026-08-18

**Authors:** Velizar Shivarov

**Affiliations:** 1 Department of Experimental Research, Medical University Pleven, 5800 Pleven, Bulgaria; vshivarov@abv.bg; 2 Department of Clinical Hematology, St. Sophia General Hospital, 1618 Sofia, Bulgaria

**Keywords:** ibrutinib, chronic lymphocytic leukemia, small lymphocytic lymphoma, polypharmacy, CYP3A, antithrombotic therapy, pharmacovigilance, FAERS

## Abstract

**Background/Objectives:** Ibrutinib is an established treatment for chronic lymphocytic leukemia/small lymphocytic lymphoma (CLL/SLL), but its use commonly occurs in older patients with polypharmacy, cardiovascular comorbidity and infection vulnerability. CYP3A-modifying drugs and antithrombotic agents are clinically actionable co-exposure domains because they may alter ibrutinib exposure, bleeding risk or the management of atrial arrhythmia. This study evaluated post-marketing reporting patterns for these co-exposures in FAERS. **Methods:** Adult FAERS reports through 25Q2 with ibrutinib exposure and CLL/SLL-compatible indications were analyzed after false-positive indication captures were removed. Co-exposures were defined at report level using prespecified drug dictionaries, and grouped adverse-event reporting was evaluated using reporting odds ratio, proportional reporting ratio, chi-square and an information-component-style two-by-two metric. **Results:** The final cohort comprised 20,878 adult CLL/SLL + ibrutinib reports. CYP3A modifier co-exposure was present in 1373 reports (6.6%), and antithrombotic co-exposure in 3701 reports (17.7%). Antithrombotic exposure increased with age, from 9.8% in reports aged 18–64 years to 23.2% in reports aged ≥75 years. CYP3A modifier exposure was associated with enriched reporting of infection and cytopenia phenotypes, particularly severe infection-like events with CYP3A inhibitors and inducers. Antithrombotic exposure showed consistent reporting enrichment for atrial arrhythmia, bleeding and major bleeding-like events, with the strongest grouped signals among anticoagulant-exposed reports. Overlap analyses showed that the co-exposure domains were not mutually exclusive. **Conclusions:** These findings should be interpreted as reporting disproportionality rather than incidence or causal risk, but they support prioritizing CYP3A-modifying drugs and antithrombotic therapy during medication reconciliation, interaction screening and risk mitigation in older patients receiving ibrutinib.

## 1. Introduction

Ibrutinib, an irreversible inhibitor of Bruton tyrosine kinase, has transformed the treatment of CLL/SLL and remains clinically relevant despite the expanding use of newer BTK inhibitors [1,2,3]. However, its long-term administration creates a substantial opportunity for clinically relevant co-medication exposure. This is particularly important in older patients with CLL/SLL, where cardiovascular disease, infectious complications, polypharmacy and antithrombotic indications are common [4].

Two co-exposure domains are especially important. First, ibrutinib is metabolized predominantly through CYP3A, and prescribing information recommends avoiding strong CYP3A inducers and modifying management with moderate or strong CYP3A inhibitors [5,6]. Second, ibrutinib has clinically recognized bleeding toxicity and platelet-function effects; concomitant anticoagulant or antiplatelet treatment may increase hemorrhagic risk [7,8,9]. Atrial fibrillation adds further complexity because it may create an indication for anticoagulation in the same patients in whom bleeding risk is increased [10,11].

The clinical relevance of polypharmacy in CLL/SLL is increasing as treatment duration lengthens and patients remain on continuous targeted therapy for years rather than discrete courses of chemoimmunotherapy. A single medication list may contain long-term cardiovascular drugs, intermittent antimicrobials, gastric-protective agents, analgesics, herbal preparations and antithrombotic therapy. For a drug such as ibrutinib, these background medications are not merely comorbidity markers; they may influence exposure, toxicity recognition, dose interruption and downstream treatment decisions. Therefore, the medication context around ibrutinib is an important component of real-world drug safety.

CYP3A interactions are a particularly direct example of this problem. Ibrutinib undergoes extensive CYP3A-mediated metabolism; therefore, inhibitors can increase exposure and potentially amplify concentration-related toxicity, while inducers can reduce exposure and may contribute to treatment failure or complex clinical deterioration. The clinical management of these interactions is usually straightforward in principle, but difficult in practice because strong or moderate inhibitors may be prescribed transiently for fungal, bacterial or viral complications, and because medication histories in spontaneous reports may be incomplete. A transparent dictionary of CYP3A modifiers is therefore essential for clinical interpretability.

Antithrombotic co-exposure represents a second, mechanistically distinct drug-safety problem. Ibrutinib is associated with platelet dysfunction and bleeding, but it can also be associated with atrial fibrillation, which may create a legitimate indication for anticoagulation. In this setting, the question is not whether all co-exposure is inappropriate. Rather, clinicians need to distinguish unavoidable, intentional co-exposure requiring monitoring from avoidable combinations such as non-essential aspirin, duplicate antithrombotic therapy or unrecognized short courses of interacting anti-infectives. This distinction is central to the present analysis and to its clinical value.

FDA Adverse Events Reporting System (FAERS) cannot define incidence, relative clinical risk or survival differences. However, it can identify post-marketing reporting patterns in large populations, including older and medically complex patients who may be underrepresented in trials. The analysis was therefore designed as a descriptive pharmacovigilance study focused on signal localization: which co-exposure domains occur frequently, which adverse-event groups are enriched, and whether the patterns differ by age and sex.

Recent population-based evidence has shown that potential drug interactions with ibrutinib are common in CLL and may be associated with clinically meaningful outcomes [4]. Spontaneous reporting systems cannot replace cohort studies, but they can provide broad post-marketing visibility into patterns of co-exposure and adverse-event reporting in populations that may be underrepresented in trials. The present study used the FAERS database to describe age- and sex-specific co-exposure to CYP3A modifiers and antithrombotic drugs among adult CLL/SLL reports with ibrutinib exposure, and to evaluate whether these co-exposures were associated with differential reporting of adverse-event groups and preferred terms [12].

From a drug-safety and polypharmacy perspective, these co-exposures represent modifiable medication-safety domains: some are clinically intentional and require monitoring, whereas others may be avoidable through structured reconciliation, interaction screening and risk mitigation.

## 2. Materials and Methods

### 2.1. Data Sources and Extraction

This was a retrospective disproportionality analysis of FAERS individual case safety reports. FAERS 25Q2 data were processed using an R workflow based on DiAna-derived import functions [13]. The analysis used report-level linkage between ibrutinib exposure and CLL/SLL-compatible indications. Duplicate handling was performed before cohort construction. When DiAna duplicate flags were available, reports flagged as duplicates were excluded; when duplicate flags were not available but CASEID was present, the workflow retained the latest numeric PRIMARYID per CASEID; otherwise, unique PRIMARYID values were used. After this step, DEMO, DRUG, REAC, OUTC, THER and INDI tables were restricted to the retained report identifiers. In addition, each exposure and each adverse-event endpoint was collapsed to a single report-level indicator so that repeated drug rows or repeated reaction rows within the same report did not increase the numerator. FAERS is a spontaneous reporting database; therefore, all estimates describe reporting patterns and should not be interpreted as incidence, prevalence, absolute risk, survival effect or causal effect [13,14].

Eligible reports were adult reports with age ≥18 years, ibrutinib or Imbruvica listed among reported drugs, and indication terms compatible with chronic lymphocytic leukemia/leukemia, CLL, small lymphocytic lymphoma or SLL. Two false-positive indication term families were excluded before cohort construction: CHRONIC LYMPHOCYTIC INFLAMMATION WITH PONTINE PERIVASCULAR ENHANCEMENT RESPONSIVE TO STEROIDS and T-CELL CHRONIC LYMPHOCYTIC LEUKAEMIA/LEUKEMIA. This exclusion step removed 230 false-positive indication rows before the final adult CLL/SLL + ibrutinib cohort was defined.

Co-exposure classes were defined at report level using DRUG role codes primary suspect, secondary suspect, concomitant or interacting. CYP3A modifier exposure comprised prespecified strong or moderate CYP3A inhibitors and CYP3A inducers; separate subanalyses evaluated any CYP3A inhibitor, strong CYP3A inhibitor, moderate CYP3A inhibitor and CYP3A inducer (Table 1; Supplementary Table S1). Antithrombotic exposure comprised anticoagulants and antiplatelet agents, with separate subanalyses for each subgroup (Supplementary Table S1). The CYP3A modifier dictionary is shown in the main article because these drugs are the most direct medication-reconciliation targets for clinicians prescribing ibrutinib. Age was grouped as 18–64, 65–74 and ≥75 years. Sex was grouped as male, female or unknown.

Reaction preferred terms were taken from the FAERS REAC table as imported through DiAna and standardized as uppercase text strings. Adverse-event groupings were created using prespecified user-defined keyword dictionaries applied to these preferred-term strings for bleeding, major bleeding-like events, infection, severe infection-like events, atrial arrhythmia, hypertension, cytopenia and diarrhea (Supplementary Table S2). These AE groups were not formal Standardized MedDRA Queries or hierarchy-derived groupings. Hypertension and diarrhea were retained as prespecified comparator AE groups because both are recognized during ibrutinib therapy but were not expected to show the same co-exposure-specific pattern as infection/cytopenia for CYP3A modifiers or atrial arrhythmia/bleeding for antithrombotic agents.

### 2.2. Formal Analysis

For each co-exposure analysis, reports with the co-exposure were compared with adult CLL/SLL + ibrutinib reports without that co-exposure. Disproportionality was quantified using reporting odds ratio (ROR) with 95% confidence interval, proportional reporting ratio (PRR), chi-square and an information-component-style two-by-two metric with 95% confidence interval. For a 2 × 2 table, a represented exposed reports with the AE, b exposed reports without the AE, c comparator reports with the AE, and d comparator reports without the AE. A Haldane–Anscombe continuity correction of 0.5 was applied when any cell was zero. The ROR was calculated as (a × d)/(b × c), and PRR as [a/(a + b)]/[c/(c + d)]. The IC-style metric was calculated as log2[(a/N)/(((a + b)/N) × ((a + c)/N))], with a normal-approximation standard error used to derive IC025 and IC975. This metric was used as a transparent two-by-two information component rather than a full Bayesian shrinkage implementation. A grouped signal was considered more robust when supported by multiple criteria: ROR025 > 1 with at least three exposed cases, PRR ≥ 2 with chi-square ≥ 4 and at least three exposed cases, and IC025 > 0 with at least three exposed cases [15,16,17]. Because all three metrics are derived from the same underlying 2 × 2 table, agreement across criteria should be interpreted as concordance of related disproportionality summaries rather than independent validation.

Prespecified AE-group analyses were considered the primary interpretive layer because they grouped clinically related reaction preferred terms and reduced the interpretive instability of sparse single-term signals. PT-level analyses were performed as exploratory screening analyses across 2197 preferred terms per exposure analysis. No false-discovery-rate or family-wise multiplicity correction was applied to the PT-level sweep; therefore, PT-level counts and rankings are reported as hypothesis-generating outputs only and were not used as the principal basis for clinical interpretation.

Exposure co-occurrence was evaluated descriptively by constructing a report-level overlap matrix across CYP3A modifier, CYP3A inhibitor, strong CYP3A inhibitor, moderate CYP3A inhibitor, CYP3A inducer, antithrombotic, anticoagulant and antiplatelet indicators. Exploratory seriousness analyses were performed for death and hospitalization reporting. The originally reported models were univariable. In response to peer review, additional joint exploratory logistic models were added for death and hospitalization reporting, including CYP3A modifier co-exposure, antithrombotic co-exposure, age group, sex and diagnosis group. These models were intended to describe whether the two main co-exposure domains retained associations with seriousness reporting after mutual adjustment and basic demographic adjustment; they were not designed to estimate causal clinical risk.

Analyses were performed in R version 4.5.2 under Windows 10. The main packages used in the workflow included *DiAna* 2.1.0, *data.table* 1.18.2.1, *dplyr* 1.2.0, *stringr* 1.6.0, *lubridate* 1.9.5, *broom* 1.0.12 and *ggplot2* 4.0.2.

## 3. Results

The final cohort included 20,878 adult reports after duplicate filtering and removal of false-positive indication captures (Supplementary Table S3). Most reports were classified as CLL (19,999, 95.8%), with smaller subsets classified as SLL (705, 3.4%) or carrying both CLL and SLL terms (174, 0.8%). The cohort included 12,913 male reports (61.8%), 7779 female reports (37.3%) and 186 reports with unknown sex (0.9%). Age distribution was broad: 5621 reports (26.9%) were in the 18–64-year group, 7337 (35.1%) in the 65–74-year group and 7920 (37.9%) in the ≥75-year group (Table 2; Supplementary Table S4).

CYP3A modifier co-exposure was present in 1373 reports (6.6%). CYP3A inhibitor co-exposure was more frequent than inducer co-exposure (1075 reports, 5.1% versus 367 reports, 1.8%). Strong and moderate CYP3A inhibitor co-exposure occurred in 242 (1.2%) and 862 (4.1%) reports, respectively. The inhibitor and inducer categories were not mutually exclusive: 69 reports contained both a CYP3A inhibitor and a CYP3A inducer term. Antithrombotic co-exposure was more common, occurring in 3701 reports (17.7%), including 1670 anticoagulant-exposed reports (8.0%) and 2382 antiplatelet-exposed reports (11.4%). CYP3A modifier and antithrombotic co-exposures also overlapped; 528 reports had both, including 318 reports with CYP3A modifier plus anticoagulant co-exposure and 281 reports with CYP3A modifier plus antiplatelet co-exposure (Supplementary Table S14). Antithrombotic co-exposure increased markedly with age, from 9.8% in reports aged 18–64 years to 23.2% in reports aged ≥75 years, while CYP3A modifier exposure remained comparatively stable across age groups (6.4%, 7.2% and 6.1% in the 18–64, 65–74 and ≥75-year groups, respectively) (Table 2; Supplementary Tables S5–S7).

**Figure 1:**
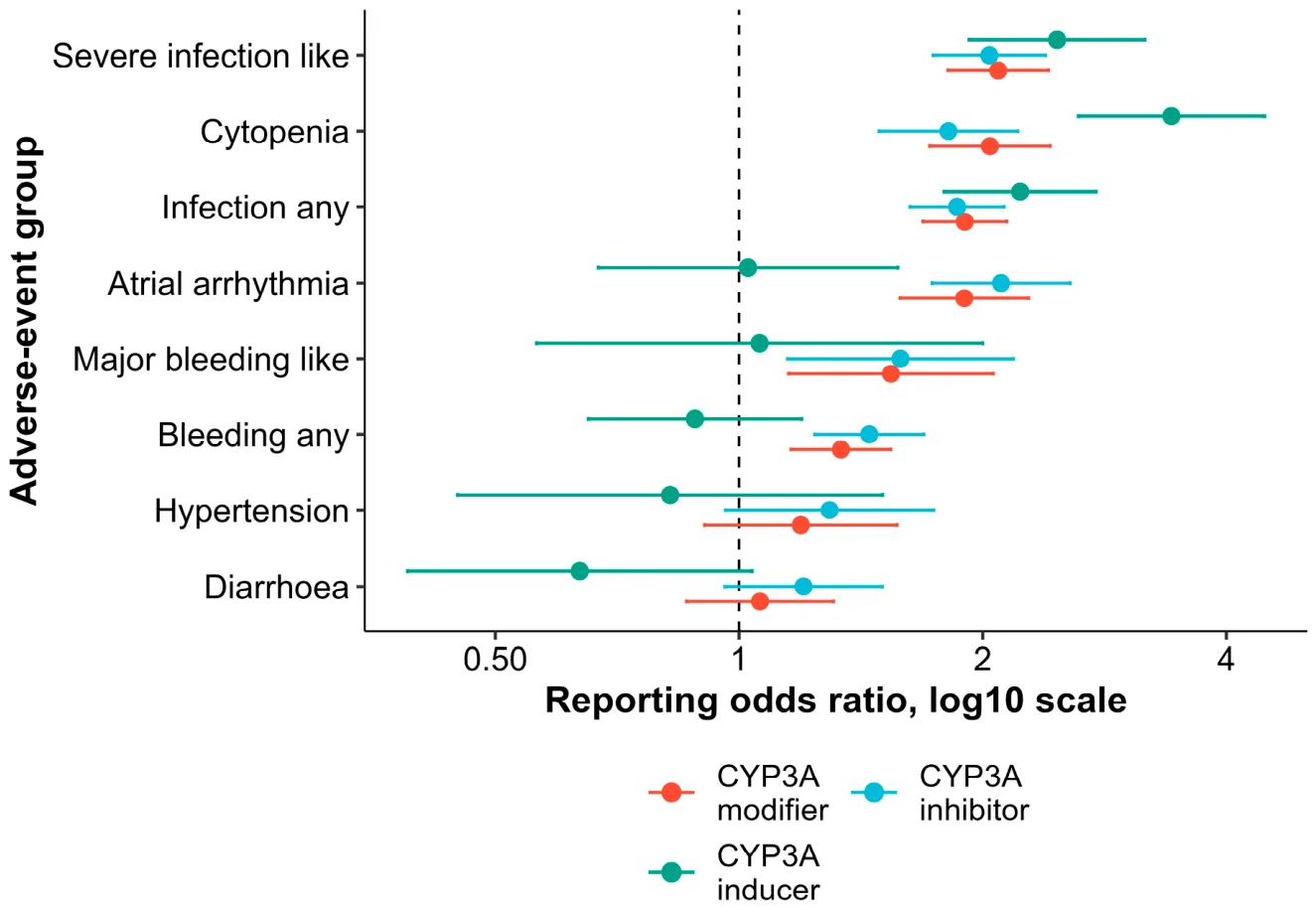
AE-group reporting odds ratios for CYP3A modifier, CYP3A inhibitor and CYP3A inducer co-exposure among adult CLL/SLL + ibrutinib reports. RORs are shown on a log10 scale; error bars represent 95% confidence intervals.

In grouped AE analyses, CYP3A modifier co-exposure was associated with higher reporting of infection-related and cytopenia phenotypes. Any CYP3A modifier exposure showed disproportional reporting for infection-any (a = 432, ROR 1.90 (1.69–2.14)) and severe infection-like events (a = 260, ROR 2.09 (1.81–2.41)), as well as cytopenia (a = 168, ROR 2.04 (1.72–2.42)). CYP3A inducer exposure showed signals supported by all three criteria for cytopenia (a = 71, ROR 3.42 (2.62–4.46)) and severe infection-like events (a = 82, ROR 2.47 (1.93–3.17)) (Table 3; Figure 1; Supplementary Tables S8–S11). By contrast, diarrhea and hypertension did not show consistent CYP3A-modifier signals across the main CYP3A analyses, supporting their role as clinically relevant but comparatively non-dominant comparator AE groups in this dataset.

**Figure 2:**
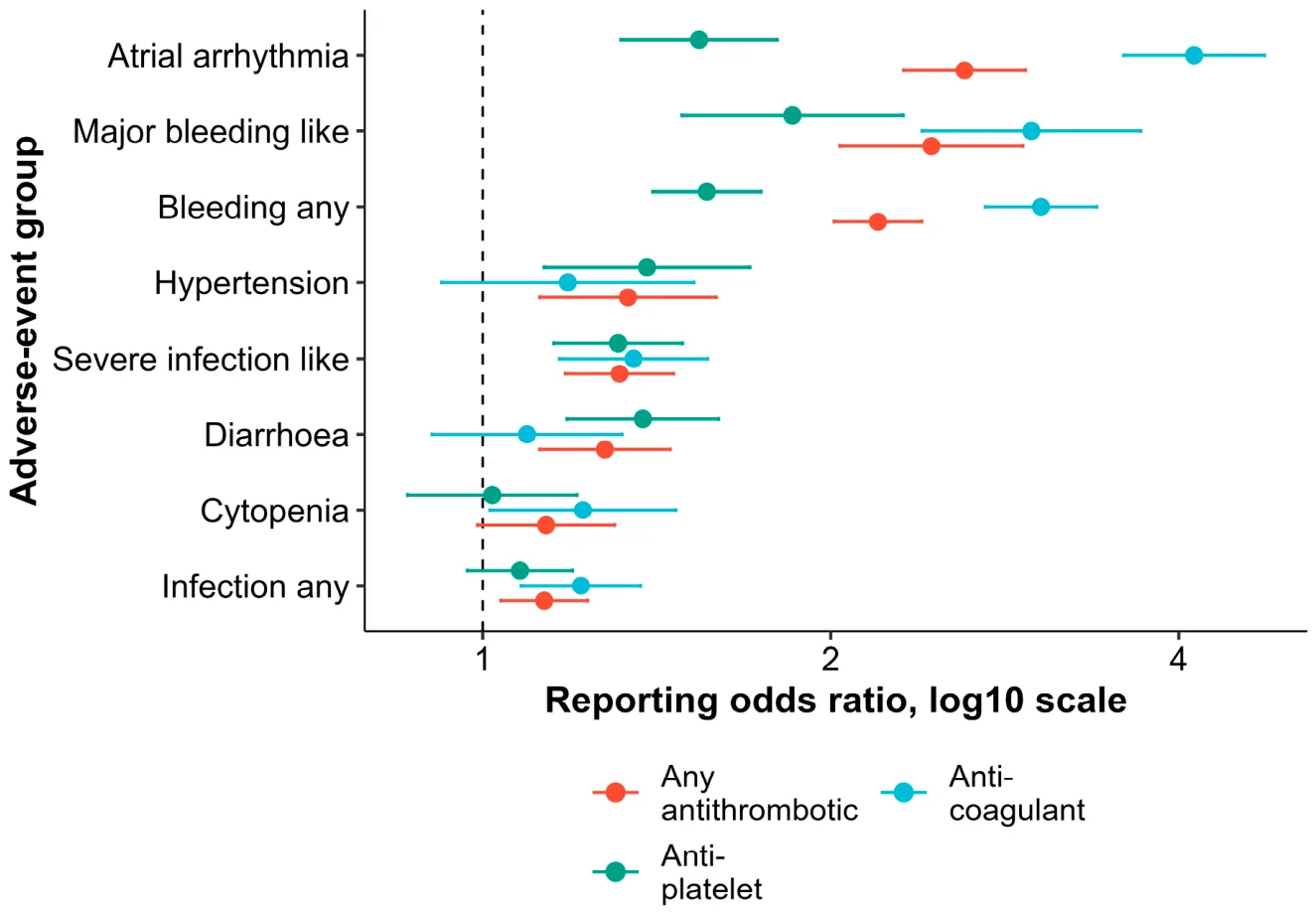
AE-group reporting odds ratios for antithrombotic, anticoagulant and antiplatelet co-exposure among adult CLL/SLL + ibrutinib reports. RORs are shown on a log10 scale; error bars represent 95% confidence intervals.

Antithrombotic co-exposure showed a distinct pattern enriched for atrial arrhythmia and bleeding-related reporting. Any antithrombotic exposure was associated with atrial arrhythmia (a = 438, ROR 2.61 (2.31–2.95)) and major bleeding-like events (a = 182, ROR 2.44 (2.04–2.93)), both supported by all three criteria. Bleeding-any was also enriched (a = 902, ROR 2.20 (2.01–2.40)). Anticoagulant co-exposure showed the strongest grouped signals, including atrial arrhythmia (a = 302, ROR 4.12 (3.58–4.74)), bleeding-any (a = 533, ROR 3.04 (2.72–3.40)) and major bleeding-like events (a = 107, ROR 2.98 (2.40–3.71)), each positive by all three criteria (Table 3; Figure 2; Supplementary Tables S8–S11). Hypertension and diarrhea were present in the antithrombotic analyses but were less central to the main clinical interpretation than atrial arrhythmia and bleeding-related groups.

**Figure 3:**
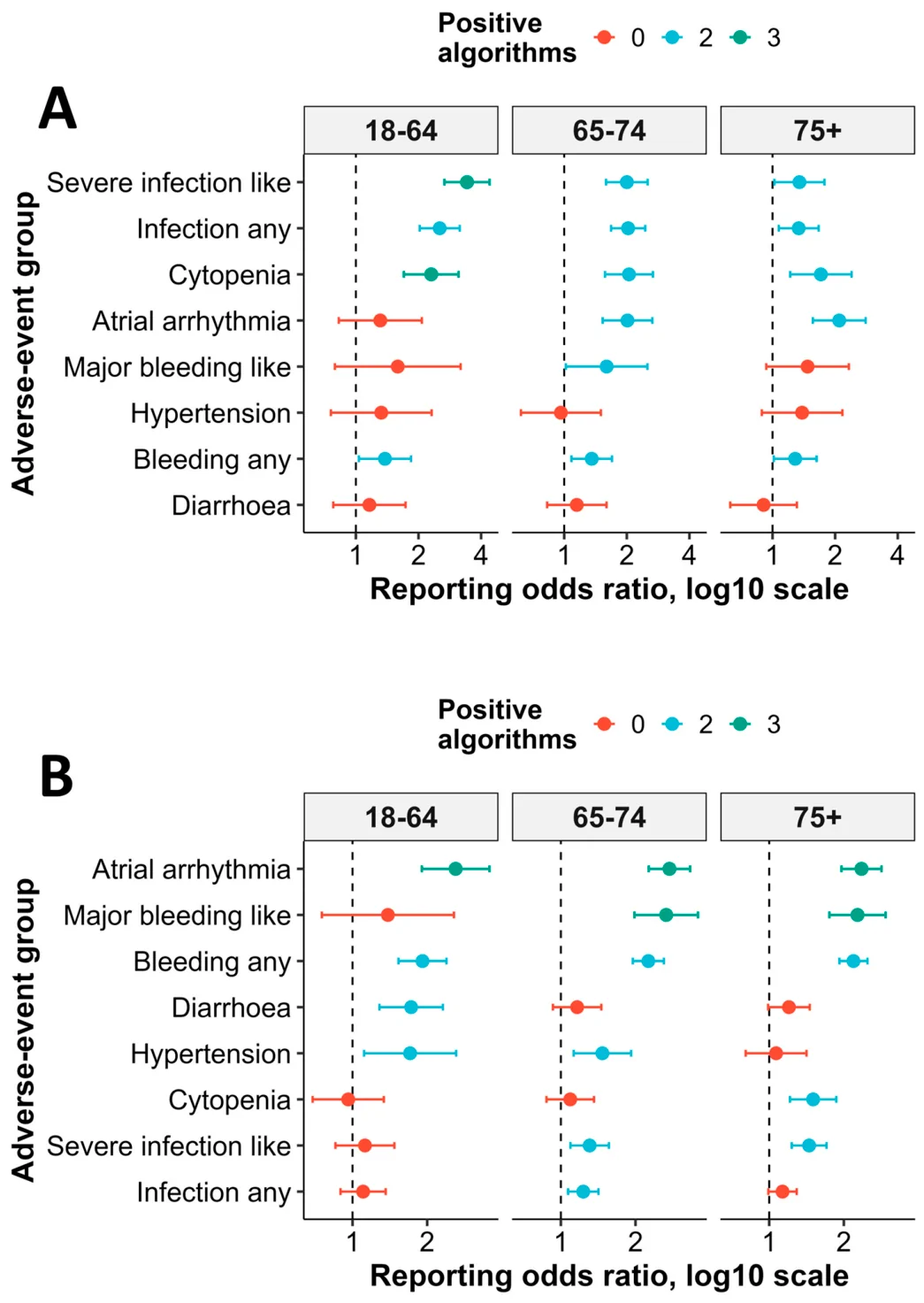
Exploratory age-stratified AE-group reporting profiles for primary co-exposure classes. (**A**) RORs for age-stratified analysis of co-exposure with CYP3A modifier. (**B**) RORs for age-stratified analysis of co-exposure with antithrombotic agent. RORs are shown on a log10 scale; error bars represent 95% confidence intervals.

**Figure 4:**
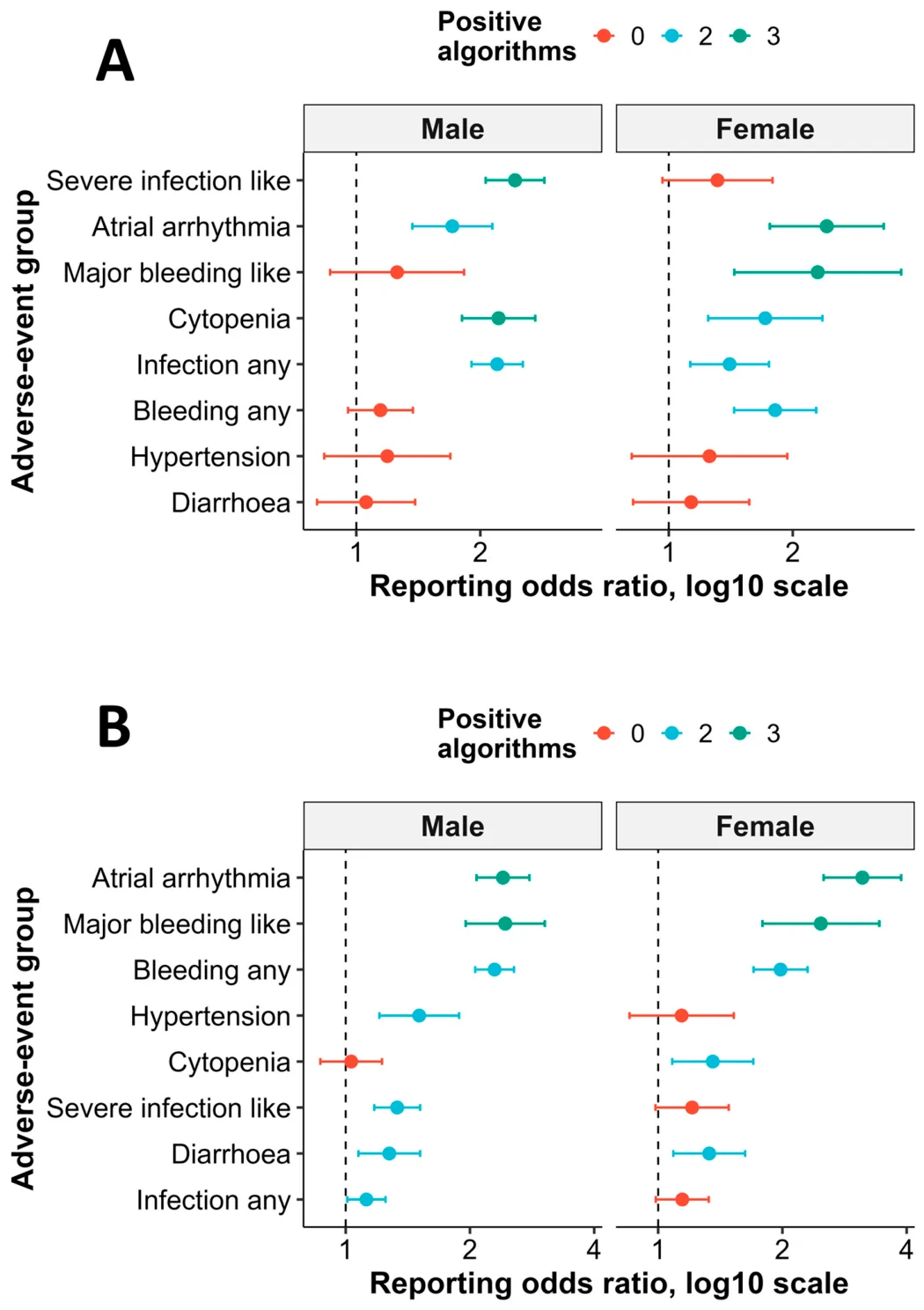
Exploratory sex-stratified AE-group reporting profiles for primary co-exposure classes. (**A**) RORs for sex-stratified analysis of co-exposure with CYP3A modifier. (**B**) RORs for sex-stratified analysis of co-exposure with antithrombotic agent. RORs are shown on a log10 scale; error bars represent 95% confidence intervals.

Age- and sex-stratified AE-group analyses showed that the main grouped signal patterns were broadly retained across clinically relevant subgroups, although the relative strength of individual signals varied by stratum and formal interaction testing was not performed (Figure 3 and Figure 4; Supplementary Tables S9 and S10). In the age-stratified analysis, CYP3A modifier co-exposure showed prominent infection- and cytopenia-related reporting in younger and intermediate-age strata, with severe infection-like events and cytopenia particularly evident among reports aged 18–64 years and infection/cytopenia signals remaining detectable in older strata. Antithrombotic co-exposure showed a more consistently age-spanning pattern of atrial arrhythmia and bleeding-related reporting, which is clinically relevant because antithrombotic co-exposure was most frequent in the ≥75-year group. In the sex-stratified analysis, CYP3A modifier co-exposure showed infection/cytopenia enrichment particularly among male reports, whereas female reports showed relatively stronger atrial arrhythmia, major bleeding-like and bleeding-any signals. Antithrombotic-associated atrial arrhythmia and bleeding-related signals were observed in both male and female strata. These sex-stratified findings should not be interpreted as definitive sex differences, because the analysis was descriptive and was not powered or modeled as a formal test of sex-by-exposure interaction. Nevertheless, the figures help localize the overall pharmacovigilance signal to clinically recognizable strata in which medication review and monitoring may be especially relevant.

In complementary PT-level analyses, 2197 preferred terms were tested per exposure analysis. The number of PTs positive by all three criteria ranged from 75 for CYP3A inducer exposure to 209 for any CYP3A modifier exposure. Because no multiplicity correction was applied to this exploratory PT-level sweep, these counts should be interpreted only as an uncorrected screening description. Many high-ROR PT-level signals were sparse and sometimes reflected drug-interaction coding, infection subtypes, coagulation monitoring terms or comorbidity markers. For this reason, the main interpretation emphasizes prespecified AE-group analyses, and PT-level rankings are provided in Supplementary Table S12.

Exploratory univariable reporting models for seriousness outcomes were performed with the intention to describe whether selected co-exposure and demographic variables were associated with reporting of death or hospitalization within the adult CLL/SLL + ibrutinib FAERS cohort (Supplementary Table S13). Death reporting was higher among reports with CYP3A modifier co-exposure (OR 1.38, 95% CI 1.19–1.58) and was particularly increased for CYP3A inducer co-exposure (OR 1.98, 95% CI 1.56–2.51), whereas CYP3A inhibitor co-exposure was not significantly associated with death reporting (OR 1.14, 95% CI 0.97–1.34). Death reporting was also higher in older age groups, especially among reports from patients aged ≥75 years (OR 1.79, 95% CI 1.62–1.98), and among male reports (OR 1.34, 95% CI 1.24–1.46). In contrast, antithrombotic and antiplatelet co-exposure showed lower odds of death reporting in univariable models, an observation that should be interpreted cautiously because reporting of death in FAERS is strongly influenced by indication, comorbidity, treatment context and reporting behavior. Hospitalization reporting showed a more consistent association with co-exposure. CYP3A modifier co-exposure was associated with increased hospitalization reporting (OR 1.55, 95% CI 1.38–1.73), with similar associations for CYP3A inhibitors (OR 1.58, 95% CI 1.39–1.78) and CYP3A inducers (OR 1.51, 95% CI 1.22–1.86). Antithrombotic co-exposure was also associated with increased hospitalization reporting (OR 1.64, 95% CI 1.53–1.76), particularly anticoagulant co-exposure (OR 1.80, 95% CI 1.63–2.00), with a weaker but still evident association for antiplatelet co-exposure (OR 1.52, 95% CI 1.39–1.66). In joint exploratory models including CYP3A modifier co-exposure, antithrombotic co-exposure, age group, sex and diagnosis group, CYP3A modifier co-exposure remained associated with death reporting (adjusted OR 1.48, 95% CI 1.28–1.71) and hospitalization reporting (adjusted OR 1.40, 95% CI 1.25–1.57). Antithrombotic co-exposure remained associated with hospitalization reporting (adjusted OR 1.57, 95% CI 1.46–1.69) but showed lower death reporting after adjustment (adjusted OR 0.74, 95% CI 0.67–0.83) (Supplementary Table S15). These models support the interpretation that seriousness reporting is strongly shaped by co-exposure domain, age, sex, diagnosis group and reporting context, but they remain descriptive and cannot adjust for comorbidity burden, disease severity, dose, treatment duration or temporal sequence.

## 4. Discussion

The present revision emphasizes the drug-safety and polypharmacy implications of the analysis. The principal clinical question is not whether FAERS can provide representative incidence estimates; it cannot. Rather, the question is whether a large spontaneous-reporting database can help identify medication combinations and AE domains that repeatedly appear together in post-marketing reports and that are plausible targets for medication-safety interventions. In this respect, the study is intended to complement, not replace, cohort analyses and clinical pharmacology studies.

This FAERS analysis identified substantial co-medication exposure among adult CLL/SLL reports with ibrutinib. Antithrombotic co-exposure was substantially more common than CYP3A modifier exposure and increased strongly with age, consistent with the clinical reality that older CLL/SLL patients often have competing cardiovascular and thromboembolic indications. CYP3A modifier exposure was less frequent overall but remained clinically important because both inhibitors and inducers can plausibly alter ibrutinib exposure or treatment effectiveness. The newly included CYP3A modifier table in the main article is intended to make this result more usable for clinicians: azole antifungals, macrolides, selected antivirals, calcium-channel blockers, amiodarone and enzyme-inducing anticonvulsants or antimicrobials are practical medication-list items that can be checked at prescribing, admission, discharge and antimicrobial-review time points.

The grouped AE results suggest two complementary safety patterns. CYP3A modifier exposure was most consistently associated with infection-related and cytopenia reporting, while CYP3A inducer exposure showed particularly strong signals for cytopenia and severe infection-like events. These findings should not be interpreted as proving pharmacokinetic causality, because FAERS cannot distinguish dose adjustment, treatment interruption, indication severity, immunosuppression, antimicrobial prophylaxis or confounding by co-treatment. The CYP3A inducer findings are also clinically complex because some inducer terms may identify patients with other comorbidities, infection histories or interacting drug regimens rather than a simple exposure-response effect. Nevertheless, the pattern is compatible with the need for careful review of interacting medications and infectious risk in ibrutinib-treated CLL/SLL patients [4,5].

The antithrombotic findings are clinically intuitive and potentially more actionable. Any antithrombotic exposure was associated with enriched reporting of atrial arrhythmia and bleeding phenotypes, and anticoagulant-exposed reports showed the strongest grouped signals for atrial arrhythmia, bleeding and major bleeding-like events. This probably reflects a complex clinical loop: ibrutinib increases the risk of atrial fibrillation in susceptible patients, atrial fibrillation may prompt anticoagulation, and anticoagulation may amplify the consequences of ibrutinib-associated platelet dysfunction. FAERS cannot resolve the temporal order, but the reporting pattern reinforces existing recommendations to individualize anticoagulant and antiplatelet decisions in ibrutinib-treated patients [7,8,10]. It also explains why the analysis may be useful to clinicians: it highlights the specific medication classes and AE domains that should be reviewed together rather than in isolation.

The age-stratified findings should be interpreted in light of the expected clinical gradient in comorbidity. Older people are more likely to have atrial fibrillation, vascular disease, renal impairment, prior thromboembolism, hypertension and exposure to interacting drugs. Therefore, the higher frequency of antithrombotic co-exposure in the ≥75-year group should not be read as evidence of inappropriate prescribing or as a direct explanation of survival. It more likely identifies a subgroup with greater cardiovascular complexity and a higher baseline probability of both medication exposure and serious clinical outcomes. The value of the signal is therefore practical rather than etiologic: older ibrutinib-treated patients are the group in whom medication reconciliation, bleeding-risk review and rhythm surveillance are most likely to find clinically relevant issues.

The sex-stratified analysis is more exploratory. Some patterns differed visually between male and female reports, including relatively stronger infection/cytopenia enrichment among male reports with CYP3A modifier co-exposure and relatively stronger bleeding/atrial-arrhythmia patterns among female reports in some strata. However, the study did not test formal sex-by-exposure interactions and should not be presented as demonstrating statistically significant sex differences. The more objective conclusion is that the main antithrombotic-associated atrial arrhythmia and bleeding signals were observed in both male and female reports, while possible sex-specific differences require confirmation in datasets with denominators, treatment duration and clinical covariates.

The clinical implications should therefore be framed as general medication-safety guidance supported by existing prescribing information and clinical literature, not as management rules derived from FAERS alone. Before and during ibrutinib treatment, clinicians can reasonably prioritize review of CYP3A inhibitors and inducers, antiplatelet drugs, anticoagulants, herbal preparations and temporary anti-infective prescriptions. Where strong or moderate CYP3A inhibition is unavoidable, product-label guidance on interruption or dose modification should be considered; strong CYP3A induction should generally be avoided when possible because of the potential for reduced ibrutinib exposure. For patients requiring anticoagulation or antiplatelet therapy, the present data support attention to indication strength, unnecessary aspirin exposure, duplicate antithrombotic therapy, mucosal or gastrointestinal bleeding symptoms, blood counts, renal function and peri-procedural planning. These steps are not novel treatment recommendations, but they translate the pharmacovigilance pattern into a pragmatic workflow: identify high-risk co-exposure, classify it as intentional or avoidable, mitigate modifiable contributors, and monitor the patients in whom co-exposure is most concentrated [18,19,20,21].

Several limitations are inherent to FAERS and are central to interpretation. Reports are spontaneous and subject to under-reporting, duplicate or incomplete information, stimulated reporting, variable coding quality and missing clinical denominators [13,14,15]. Although the workflow applied duplicate filtering before cohort construction and collapsed exposure and AE indicators to the report level, residual duplicate submissions across different identifiers cannot be completely excluded. FAERS is also not representative of treated CLL/SLL populations: older patients may be sicker, may use more medications, may have more complications requiring additional therapy and may be reported differently from younger patients. Therefore, the findings cannot estimate incidence, prevalence, survival, absolute risk or causal effects. Drug exposure does not prove actual administration, and report-level co-exposure analysis cannot establish temporal sequence, dose, duration, pharmacokinetic interaction, or causality [12,22]. The CYP3A modifier and antithrombotic domains overlapped in a subset of reports, and although an overlap matrix and exploratory joint seriousness models were added, the analysis still cannot fully separate correlated polypharmacy from disease severity, frailty, cardiovascular comorbidity or reporting behavior. PT-level analyses were uncorrected for multiple testing and are hypothesis-generating only. Finally, the comparator was restricted to adult CLL/SLL + ibrutinib reports without the co-exposure of interest; this strengthens within-therapy interpretability but does not eliminate confounding by comorbidity, disease severity or treatment context [12,22,23].

## 5. Conclusions

Among adult FAERS reports with CLL/SLL and ibrutinib exposure, antithrombotic co-exposure was common and strongly age-associated, while CYP3A modifier co-exposure was less frequent but clinically relevant. Grouped disproportionality analyses highlighted infection/cytopenia patterns with CYP3A modifiers and atrial arrhythmia/bleeding patterns with antithrombotics, especially anticoagulants. The study does not provide representative incidence estimates or causal risk estimates, but it identifies medication classes and AE domains that are clinically actionable during medication reconciliation. These findings support risk-focused review of CYP3A-modifying drugs and antithrombotic therapy in older CLL/SLL patients receiving ibrutinib and provide a basis for confirmatory analyses in longitudinal clinical datasets.

## Figures and Tables

**Table 1:** CYP3A modifier drug/substance dictionary used in the main exposure definition. The complete co-medication dictionary, including antithrombotic agents, is provided in Supplementary Table S1.

CYP3A Modifier Component	Standardized Drug/Substance Terms
Strong CYP3A inhibitor	KETOCONAZOLE; ITRACONAZOLE; POSACONAZOLE; VORICONAZOLE; CLARITHROMYCIN; TELITHROMYCIN; NEFAZODONE; RITONAVIR; COBICISTAT; INDINAVIR; NELFINAVIR; SAQUINAVIR; LOPINAVIR; BOCEPREVIR; TELAPREVIR
Moderate CYP3A inhibitor	FLUCONAZOLE; ERYTHROMYCIN; DILTIAZEM; VERAPAMIL; CIPROFLOXACIN; APREPITANT; FOSAPREPITANT; AMIODARONE; DRONEDARONE; IMATINIB; CRIZOTINIB; ATAZANAVIR; DARUNAVIR; AMPRENAVIR; FOSAMPRENAVIR
CYP3A inducer	RIFAMPICIN; RIFAMPIN; RIFABUTIN; RIFAPENTINE; CARBAMAZEPINE; PHENYTOIN; PHENOBARBITAL; PHENOBARBITONE; PRIMIDONE; EFAVIRENZ; NEVIRAPINE; ETRAVIRINE; BOSENTAN; MODAFINIL; ENZALUTAMIDE; APALUTAMIDE; MITOTANE; DEXAMETHASONE; ST JOHN’S WORT; HYPERICUM PERFORATUM

**Table 2:** Major cohort and comparator-group characteristics. Abbreviations: CLL, chronic lymphocytic leukemia/leukaemia; SLL, small lymphocytic lymphoma; CYP3A, cytochrome P450 3A.

Characteristic	Overall Adult CLL/SLL + Ibrutinib Cohort	CYP3A Modifier Co-Exposure	Comparator: No CYP3A Modifier Co-Exposure	Antithrombotic Co-Exposure	Comparator: No Antithrombotic Co-Exposure
Total reports, n	20,878	1373	19,505	3701	17,177
Age group					
18–64 years, n (%)	5621 (26.9%)	362 (26.4%)	5259 (27.0%)	551 (14.9%)	5070 (29.5%)
65–74 years, n (%)	7337 (35.1%)	531 (38.7%)	6806 (34.9%)	1310 (35.4%)	6027 (35.1%)
≥75 years, n (%)	7920 (37.9%)	480 (35.0%)	7440 (38.1%)	1840 (49.7%)	6080 (35.4%)
Age unknown, n (%)	0 (0.0%)	0 (0.0%)	0 (0.0%)	0 (0.0%)	0 (0.0%)
Sex					
Male, n (%)	12,913 (61.8%)	907 (66.1%)	12,006 (61.6%)	2423 (65.5%)	10,490 (61.1%)
Female, n (%)	7779 (37.3%)	452 (32.9%)	7327 (37.6%)	1259 (34.0%)	6520 (38.0%)
Unknown, n (%)	186 (0.9%)	14 (1.0%)	172 (0.9%)	19 (0.5%)	167 (1.0%)
Indication					
CLL, n (%)	19,999 (95.8%)	1312 (95.6%)	18,687 (95.8%)	3527 (95.3%)	16,472 (95.9%)
SLL, n (%)	705 (3.4%)	48 (3.5%)	657 (3.4%)	133 (3.6%)	572 (3.3%)
Both CLL and SLL terms, n (%)	174 (0.8%)	13 (0.9%)	161 (0.8%)	41 (1.1%)	133 (0.8%)
Outcomes					
Death, n (%)	3127 (15.0%)	263 (19.2%)	2864 (14.7%)	499 (13.5%)	2628 (15.3%)
Hospitalization, n (%)	9624 (46.1%)	772 (56.2%)	8852 (45.4%)	2082 (56.3%)	7542 (43.9%)
Life-threatening, n (%)	786 (3.8%)	102 (7.4%)	684 (3.5%)	173 (4.7%)	613 (3.6%)

**Table 3:** Selected AE-group disproportionality results. Positive algorithms indicate how many of ROR, PRR and IC criteria were positive.

Exposure Analysis	AE Group	Exposed with AE	Comparator with AE	ROR (95% CI)	IC025	Positive Algorithms
Any CYP3A modifier	Infection any	432	3795	1.90 (1.69–2.14)	0.53	2
Any CYP3A modifier	Severe infection like	260	1961	2.09 (1.81–2.41)	0.68	2
Any CYP3A modifier	Cytopenia	168	1247	2.04 (1.72–2.42)	0.66	2
Any CYP3A modifier	Atrial arrhythmia	144	1134	1.90 (1.58–2.28)	0.57	2
Any CYP3A modifier	Major bleeding like	52	486	1.54 (1.15–2.06)	0.19	2
CYP3A inhibitor	Infection any	336	3891	1.86 (1.63–2.13)	0.50	2
CYP3A inhibitor	Severe infection like	202	2019	2.04 (1.74–2.39)	0.65	2
CYP3A inhibitor	Atrial arrhythmia	124	1154	2.11 (1.73–2.56)	0.69	2
CYP3A inducer	Cytopenia	71	1344	3.42 (2.62–4.46)	1.22	3
CYP3A inducer	Severe infection like	82	2139	2.47 (1.93–3.17)	0.80	3
Any antithrombotic	Atrial arrhythmia	438	840	2.61 (2.31–2.95)	0.85	3
Any antithrombotic	Major bleeding like	182	356	2.44 (2.04–2.93)	0.77	3
Any antithrombotic	Bleeding any	902	2197	2.20 (2.01–2.40)	0.65	2
Any antithrombotic	Severe infection like	478	1743	1.31 (1.18–1.46)	0.17	2
Anticoagulant	Atrial arrhythmia	302	976	4.12 (3.58–4.74)	1.44	3
Anticoagulant	Bleeding any	533	2566	3.04 (2.72–3.40)	1.01	3
Anticoagulant	Major bleeding like	107	431	2.98 (2.40–3.71)	1.08	3
Antiplatelet	Major bleeding like	102	436	1.85 (1.49–2.31)	0.49	2
Antiplatelet	Bleeding any	487	2612	1.56 (1.40–1.74)	0.36	2
Antiplatelet	Atrial arrhythmia	206	1072	1.54 (1.32–1.80)	0.33	2

## Data Availability

The source data are publicly available from the FDA Adverse Event Reporting System. Processed data and analysis code are available from the corresponding author upon reasonable request.

## Supplementary Materials

The following supporting information can be downloaded at: https://www.mdpi.com/article/10.3390/jcm15166370/s1. Table S1. Drug/substance terms included in each co-medication dictionary. Table S2. AE-group preferred-term dictionary. Table S3. Final analysis summary. Table S4. Cohort distribution by diagnosis, age group and sex. Table S5. Overall co-exposure summary. Table S6. Co-exposure summary by age group. Table S7. Co-exposure summary by sex. Table S8. Overall AE-group signal results for all co-exposure analyses. Table S9. Age-stratified AE-group signal results. Table S10. Sex-stratified AE-group signal results. Table S11. PT-level signal counts by exposure analysis. Table S12. Top 15 PT-level signals by exposure analysis. Table S13. Exploratory univariable death and hospitalization reporting models. Table S14. Report-level exposure co-occurrence matrix. Cells show the number of unique reports positive for both the row exposure and the column exposure. Diagonal cells show total exposed reports for each exposure indicator. Table S15. Exploratory joint logistic models for death and hospitalization reporting. Models include CYP3A modifier co-exposure, antithrombotic co-exposure, age group, sex and diagnosis group. Reference categories are no co-exposure, age 18-64 years, female sex and CLL diagnosis group. Results are descriptive and should not be interpreted as causal estimates.

## Abbreviations

The following abbreviations are used in this manuscript:

**Abbreviation**	**Definition**
AE	adverse event
CLL	chronic lymphocytic leukemia
CYP3A	cytochrome P450 3A
FAERS	FDA Adverse Event Reporting System
IC	information component
JCM	Journal of Clinical Medicine
PRR	proportional reporting ratio
PT	preferred term
ROR	reporting odds ratio
SLL	small lymphocytic lymphoma
