# Supplementary material for: Drug Safety and Polypharmacy Signals in Ibrutinib-Treated Chronic Lymphocytic Leukemia/Small Lymphocytic Lymphoma: Age- and Sex-Specific FAERS Analysis of CYP3A Modifier and Antithrombotic Co-Exposure
Source: J Clin Med. 2026 Aug 18;15(16):6370. doi: 10.3390/jcm15166370 (PMC13513224; doi:10.3390/jcm15166370)
Supplement: Supplementary file 1 [file jcm-15-06370-s001.zip › jcm-4454672-supplementary.pdf]

Supplementary Material

Drug Safety and Polypharmacy Signals in Ibrutinib-Treated Chronic Lymphocytic Leukemia/Small Lymphocytic Lymphoma: Age- and Sex-Specific FAERS Analysis of CYP3A Modifier and Antithrombotic Co-Exposure

Supplementary tables

Supplementary Table S1. Drug/substance terms included in each co-medication dictionary.

Parent domain	Component class	Standardized drug/ substance term	Exposure analyses including this term
CYP3A modifiers	Strong CYP3A inhibitor	KETOCONAZOLE	Any CYP3A modifier; CYP3A inhibitor; Strong CYP3A inhibitor
CYP3A modifiers	Strong CYP3A inhibitor	ITRACONAZOLE	Any CYP3A modifier; CYP3A inhibitor; Strong CYP3A inhibitor
CYP3A modifiers	Strong CYP3A inhibitor	POSACONAZOLE	Any CYP3A modifier; CYP3A inhibitor; Strong CYP3A inhibitor
CYP3A modifiers	Strong CYP3A inhibitor	VORICONAZOLE	Any CYP3A modifier; CYP3A inhibitor; Strong CYP3A inhibitor
CYP3A modifiers	Strong CYP3A inhibitor	CLARITHROMYCIN	Any CYP3A modifier; CYP3A inhibitor; Strong CYP3A inhibitor
CYP3A modifiers	Strong CYP3A inhibitor	TELITHROMYCIN	Any CYP3A modifier; CYP3A inhibitor; Strong CYP3A inhibitor
CYP3A modifiers	Strong CYP3A inhibitor	NEFAZODONE	Any CYP3A modifier; CYP3A inhibitor; Strong CYP3A inhibitor
CYP3A modifiers	Strong CYP3A inhibitor	RITONAVIR	Any CYP3A modifier; CYP3A inhibitor; Strong CYP3A inhibitor
CYP3A modifiers	Strong CYP3A inhibitor	COBICISTAT	Any CYP3A modifier; CYP3A inhibitor; Strong CYP3A inhibitor
CYP3A modifiers	Strong CYP3A inhibitor	INDINAVIR	Any CYP3A modifier; CYP3A inhibitor; Strong CYP3A inhibitor
CYP3A modifiers	Strong CYP3A inhibitor	NELFINAVIR	Any CYP3A modifier; CYP3A inhibitor; Strong CYP3A inhibitor
CYP3A modifiers	Strong CYP3A inhibitor	SAQUINAVIR	Any CYP3A modifier; CYP3A inhibitor; Strong CYP3A inhibitor
CYP3A modifiers	Strong CYP3A inhibitor	LOPINAVIR	Any CYP3A modifier; CYP3A inhibitor; Strong CYP3A inhibitor
CYP3A modifiers	Strong CYP3A inhibitor	BOCEPREVIR	Any CYP3A modifier; CYP3A inhibitor; Strong CYP3A inhibitor
CYP3A modifiers	Strong CYP3A inhibitor	TELAPREVIR	Any CYP3A modifier; CYP3A inhibitor; Strong CYP3A inhibitor
CYP3A modifiers	Moderate CYP3A inhibitor	FLUCONAZOLE	Any CYP3A modifier; CYP3A inhibitor; Moderate CYP3A inhibitor
CYP3A modifiers	Moderate CYP3A inhibitor	ERYTHROMYCIN	Any CYP3A modifier; CYP3A inhibitor; Moderate CYP3A inhibitor

CYP3A modifiers	Moderate CYP3A inhibitor	DILTIAZEM	Any CYP3A modifier; CYP3A inhibitor; Moderate CYP3A inhibitor
CYP3A modifiers	Moderate CYP3A inhibitor	VERAPAMIL	Any CYP3A modifier; CYP3A inhibitor; Moderate CYP3A inhibitor
CYP3A modifiers	Moderate CYP3A inhibitor	CIPROFLOXACIN	Any CYP3A modifier; CYP3A inhibitor; Moderate CYP3A inhibitor
CYP3A modifiers	Moderate CYP3A inhibitor	APREPITANT	Any CYP3A modifier; CYP3A inhibitor; Moderate CYP3A inhibitor
CYP3A modifiers	Moderate CYP3A inhibitor	FOSAPREPITANT	Any CYP3A modifier; CYP3A inhibitor; Moderate CYP3A inhibitor
CYP3A modifiers	Moderate CYP3A inhibitor	AMIODARONE	Any CYP3A modifier; CYP3A inhibitor; Moderate CYP3A inhibitor
CYP3A modifiers	Moderate CYP3A inhibitor	DRONEDARONE	Any CYP3A modifier; CYP3A inhibitor; Moderate CYP3A inhibitor
CYP3A modifiers	Moderate CYP3A inhibitor	IMATINIB	Any CYP3A modifier; CYP3A inhibitor; Moderate CYP3A inhibitor
CYP3A modifiers	Moderate CYP3A inhibitor	CRIZOTINIB	Any CYP3A modifier; CYP3A inhibitor; Moderate CYP3A inhibitor
CYP3A modifiers	Moderate CYP3A inhibitor	ATAZANAVIR	Any CYP3A modifier; CYP3A inhibitor; Moderate CYP3A inhibitor
CYP3A modifiers	Moderate CYP3A inhibitor	DARUNAVIR	Any CYP3A modifier; CYP3A inhibitor; Moderate CYP3A inhibitor
CYP3A modifiers	Moderate CYP3A inhibitor	AMPRENAVIR	Any CYP3A modifier; CYP3A inhibitor; Moderate CYP3A inhibitor
CYP3A modifiers	Moderate CYP3A inhibitor	FOSAMPRENAVIR	Any CYP3A modifier; CYP3A inhibitor; Moderate CYP3A inhibitor
CYP3A modifiers	CYP3A inducer	RIFAMPICIN	Any CYP3A modifier; CYP3A inducer
CYP3A modifiers	CYP3A inducer	RIFAMPIN	Any CYP3A modifier; CYP3A inducer
CYP3A modifiers	CYP3A inducer	RIFABUTIN	Any CYP3A modifier; CYP3A inducer
CYP3A modifiers	CYP3A inducer	RIFAPENTINE	Any CYP3A modifier; CYP3A inducer
CYP3A modifiers	CYP3A inducer	CARBAMAZEPINE	Any CYP3A modifier; CYP3A inducer
CYP3A modifiers	CYP3A inducer	PHENYTOIN	Any CYP3A modifier; CYP3A inducer
CYP3A modifiers	CYP3A inducer	PHENOBARBITAL	Any CYP3A modifier; CYP3A inducer
CYP3A modifiers	CYP3A inducer	PHENOBARBITONE	Any CYP3A modifier; CYP3A inducer
CYP3A modifiers	CYP3A inducer	PRIMIDONE	Any CYP3A modifier; CYP3A inducer
CYP3A modifiers	CYP3A inducer	EFAVIRENZ	Any CYP3A modifier; CYP3A inducer
CYP3A modifiers	CYP3A inducer	NEVIRAPINE	Any CYP3A modifier; CYP3A inducer
CYP3A modifiers	CYP3A inducer	ETRAVIRINE	Any CYP3A modifier; CYP3A inducer
CYP3A modifiers	CYP3A inducer	BOSENTAN	Any CYP3A modifier; CYP3A inducer
CYP3A modifiers	CYP3A inducer	MODAFINIL	Any CYP3A modifier; CYP3A inducer
CYP3A modifiers	CYP3A inducer	ENZALUTAMIDE	Any CYP3A modifier; CYP3A inducer
CYP3A modifiers	CYP3A inducer	APALUTAMIDE	Any CYP3A modifier; CYP3A inducer
CYP3A modifiers	CYP3A inducer	MITOTANE	Any CYP3A modifier; CYP3A inducer
CYP3A modifiers	CYP3A inducer	DEXAMETHASONE	Any CYP3A modifier; CYP3A inducer
CYP3A modifiers	CYP3A inducer	ST JOHN'S WORT	Any CYP3A modifier; CYP3A inducer
CYP3A modifiers	CYP3A inducer	HYPERICUM PERFORATUM	Any CYP3A modifier; CYP3A inducer
Antithrombotic agents	Anticoagulant	WARFARIN	Any antithrombotic; Anticoagulant

Antithrombotic agents	Anticoagulant	APIXABAN	Any antithrombotic; Anticoagulant
Antithrombotic agents	Anticoagulant	RIVAROXABAN	Any antithrombotic; Anticoagulant
Antithrombotic agents	Anticoagulant	EDOXABAN	Any antithrombotic; Anticoagulant
Antithrombotic agents	Anticoagulant	DABIGATRAN	Any antithrombotic; Anticoagulant
Antithrombotic agents	Anticoagulant	HEPARIN	Any antithrombotic; Anticoagulant
Antithrombotic agents	Anticoagulant	ENOXAPARIN	Any antithrombotic; Anticoagulant
Antithrombotic agents	Anticoagulant	DALTEPARIN	Any antithrombotic; Anticoagulant
Antithrombotic agents	Anticoagulant	TINZAPARIN	Any antithrombotic; Anticoagulant
Antithrombotic agents	Anticoagulant	FONDAPARINUX	Any antithrombotic; Anticoagulant
Antithrombotic agents	Anticoagulant	ARGATROBAN	Any antithrombotic; Anticoagulant
Antithrombotic agents	Anticoagulant	BIVALIRUDIN	Any antithrombotic; Anticoagulant
Antithrombotic agents	Antiplatelet	ASPIRIN	Any antithrombotic; Antiplatelet
Antithrombotic agents	Antiplatelet	ACETYLSALICYLIC ACID	Any antithrombotic; Antiplatelet
Antithrombotic agents	Antiplatelet	CLOPIDOGREL	Any antithrombotic; Antiplatelet
Antithrombotic agents	Antiplatelet	PRASUGREL	Any antithrombotic; Antiplatelet
Antithrombotic agents	Antiplatelet	TICAGRELOR	Any antithrombotic; Antiplatelet
Antithrombotic agents	Antiplatelet	TICLOPIDINE	Any antithrombotic; Antiplatelet
Antithrombotic agents	Antiplatelet	DIPYRIDAMOLE	Any antithrombotic; Antiplatelet
Antithrombotic agents	Antiplatelet	CILOSTAZOL	Any antithrombotic; Antiplatelet
Antithrombotic agents	Antiplatelet	ABCIXIMAB	Any antithrombotic; Antiplatelet
Antithrombotic agents	Antiplatelet	EPTIFIBATIDE	Any antithrombotic; Antiplatelet
Antithrombotic agents	Antiplatelet	TIROFIBAN	Any antithrombotic; Antiplatelet

Supplementary Table S2. AE-group preferred-term dictionary.

AE group	Number of PTs	Included preferred terms
Atrial arrhythmia	3	ATRIAL FIBRILLATION; ATRIAL FLUTTER; SUPRAVENTRICULAR TACHYCARDIA
Bleeding any	165	ABDOMINAL WALL HAEMATOMA; ABNORMAL UTERINE BLEEDING; ADRENAL HAEMORRHAGE; ANAL HAEMORRHAGE; AORTIC INTRAMURAL HAEMATOMA; ARTERIAL HAEMORRHAGE; ARTERIOVENOUS FISTULA SITE HAEMORRHAGE; BASAL GANGLIA HAEMORRHAGE; BLEEDING TIME PROLONGED; BLEEDING VARICOSE VEIN; BONE MARROW HAEMORRHAGE; BRAIN STEM HAEMORRHAGE; BREAST HAEMATOMA; BREAST HAEMORRHAGE; BRONCHIAL HAEMORRHAGE; CATHETER SITE HAEMATOMA; CATHETER SITE HAEMORRHAGE; CENTRAL NERVOUS SYSTEM HAEMORRHAGE; CEREBELLAR HAEMORRHAGE; CEREBELLAR MICROHAEMORRHAGE; CEREBRAL HAEMATOMA; CEREBRAL HAEMORRHAGE; CHEST WALL HAEMATOMA; CHOROIDAL HAEMORRHAGE; CHRONIC GASTROINTESTINAL BLEEDING; CHRONIC PIGMENTED PURPURA; COLONIC HAEMATOMA; CONJUNCTIVAL HAEMORRHAGE; DUODENAL ULCER HAEMORRHAGE; EAR HAEMORRHAGE; ECCHYMOSIS; EPISTAXIS; EXTRADURAL HAEMATOMA; EYE HAEMATOMA; EYE HAEMORRHAGE; EYELID BLEEDING; GASTRIC HAEMORRHAGE; GASTRIC ULCER HAEMORRHAGE; GASTRIC VARICES HAEMORRHAGE; GASTROINTESTINAL HAEMORRHAGE; GASTROINTESTINAL POLYP HAEMORRHAGE; GASTROINTESTINAL ULCER HAEMORRHAGE; GENITAL HAEMORRHAGE; GINGIVAL BLEEDING; GRAFT HAEMORRHAGE; HAEMATOMA; HAEMATOMA INFECTION; HAEMATOMA MUSCLE; HAEMATURIA; HAEMATURIA TRAUMATIC; HAEMORRHAGE; HAEMORRHAGE INTRACRANIAL; HAEMORRHAGE SUBCUTANEOUS; HAEMORRHAGE URINARY TRACT; HAEMORRHOIDAL HAEMORRHAGE; HEAVY MENSTRUAL BLEEDING; HENOCH-SCHONLEIN PURPURA; HEPATIC HAEMATOMA; HEPATIC HAEMORRHAGE; INCISION SITE HAEMORRHAGE; INFUSION SITE HAEMORRHAGE; INJECTION SITE HAEMATOMA; INJECTION SITE HAEMORRHAGE; INTERMENSTRUAL BLEEDING; INTERNAL HAEMORRHAGE; INTESTINAL HAEMATOMA; INTESTINAL HAEMORRHAGE; INTRA-ABDOMINAL HAEMATOMA; INTRA-ABDOMINAL HAEMORRHAGE; INTRACRANIAL HAEMATOMA; INTRACRANIAL TUMOUR HAEMORRHAGE; INTRAVENTRICULAR HAEMORRHAGE; IRIS HAEMORRHAGE; LARGE INTESTINAL HAEMORRHAGE; LIP HAEMATOMA; LIP HAEMORRHAGE; LOWER GASTROINTESTINAL HAEMORRHAGE; LYMPH NODE HAEMORRHAGE; MEDIASTINAL HAEMORRHAGE; MEDICAL DEVICE SITE HAEMORRHAGE; MELAENA; MOUTH HAEMORRHAGE; MUCOCUTANEOUS HAEMORRHAGE; MUCOSAL HAEMORRHAGE; MUSCLE HAEMORRHAGE; NAIL BED BLEEDING; OCULAR RETROBULBAR HAEMORRHAGE; OESOPHAGEAL HAEMORRHAGE; OESOPHAGEAL ULCER HAEMORRHAGE; OESOPHAGEAL VARICES HAEMORRHAGE; OPTIC NERVE SHEATH HAEMORRHAGE; PALPABLE PURPURA; PARANASAL SINUS HAEMORRHAGE; PENILE HAEMORRHAGE; PEPTIC ULCER HAEMORRHAGE; PERICARDIAL HAEMORRHAGE; PERIORBITAL HAEMATOMA; PERIORBITAL HAEMORRHAGE; PHARYNGEAL HAEMORRHAGE; PITUITARY HAEMORRHAGE; POST PROCEDURAL HAEMATOMA; POST PROCEDURAL HAEMATURIA; POST PROCEDURAL HAEMORRHAGE; POSTMENOPAUSAL HAEMORRHAGE; PROCEDURAL HAEMORRHAGE; PROSTATIC HAEMORRHAGE; PULMONARY ALVEOLAR HAEMORRHAGE; PULMONARY HAEMORRHAGE; PUNCTURE SITE HAEMORRHAGE; PURPURA; PURPURA SENILE; RECTAL HAEMORRHAGE; RENAL CYST HAEMORRHAGE; RENAL

		HAEMATOMA; RENAL HAEMORRHAGE; RESPIRATORY TRACT HAEMORRHAGE; RETINAL HAEMORRHAGE; RETROPERITONEAL HAEMATOMA; RETROPERITONEAL HAEMORRHAGE; SCLERAL HAEMORRHAGE; SCROTAL HAEMATOMA; SCROTAL HAEMORRHAGE; SKIN HAEMORRHAGE; SKIN ULCER HAEMORRHAGE; SMALL INTESTINAL HAEMORRHAGE; SOFT TISSUE HAEMORRHAGE; SPINAL CORD HAEMATOMA; SPINAL CORD HAEMORRHAGE; SPLENIC HAEMATOMA; SPLENIC HAEMORRHAGE; SPONTANEOUS HAEMATOMA; SPONTANEOUS HAEMORRHAGE; STOMA SITE HAEMORRHAGE; SUBARACHNOID HAEMATOMA; SUBARACHNOID HAEMORRHAGE; SUBCAPSULAR RENAL HAEMATOMA; SUBCAPSULAR SPLENIC HAEMATOMA; SUBCUTANEOUS HAEMATOMA; SUBDURAL HAEMATOMA; SUBDURAL HAEMORRHAGE; SUBGALEAL HAEMATOMA; TESTICULAR HAEMORRHAGE; THALAMUS HAEMORRHAGE; THROMBOCYTOPENIC PURPURA; THROMBOTIC THROMBOCYTOPENIC PURPURA; TONGUE HAEMATOMA; TONGUE HAEMORRHAGE; TONSILLAR HAEMORRHAGE; TRAUMATIC HAEMATOMA; TRAUMATIC HAEMORRHAGE; TRAUMATIC INTRACRANIAL HAEMATOMA; TRAUMATIC INTRACRANIAL HAEMORRHAGE; TUMOUR HAEMORRHAGE; ULCER HAEMORRHAGE; UPPER GASTROINTESTINAL HAEMORRHAGE; URETHRAL HAEMORRHAGE; URINARY BLADDER HAEMORRHAGE; UROGENITAL HAEMORRHAGE; UTERINE HAEMORRHAGE; VAGINAL HAEMORRHAGE; VASCULAR ACCESS SITE HAEMORRHAGE; VASCULAR PURPURA; VENOUS HAEMORRHAGE; VITREOUS HAEMORRHAGE; WOUND HAEMORRHAGE
Cytopenia	28	ANAEMIA; ANAEMIA MACROCYTIC; ANAEMIA OF CHRONIC DISEASE; ANAEMIA POSTOPERATIVE; APLASTIC ANAEMIA; AUTOIMMUNE ANAEMIA; AUTOIMMUNE HAEMOLYTIC ANAEMIA; AUTOIMMUNE NEUTROPENIA; BLOOD LOSS ANAEMIA; COLD TYPE HAEMOLYTIC ANAEMIA; COOMBS NEGATIVE HAEMOLYTIC ANAEMIA; COOMBS POSITIVE HAEMOLYTIC ANAEMIA; DEFICIENCY ANAEMIA; FEBRILE NEUTROPENIA; HAEMOLYTIC ANAEMIA; HYPOCHROMIC ANAEMIA; IMMUNE THROMBOCYTOPENIA; IRON DEFICIENCY ANAEMIA; LEUKOPENIA; LYMPHOPENIA; MICROCYTIC ANAEMIA; NEPHROGENIC ANAEMIA; NEUTROPENIA; NORMOCHROMIC NORMOCYTIC ANAEMIA; NORMOCYTIC ANAEMIA; PANCYTOPENIA; PERNICIOUS ANAEMIA; THROMBOCYTOPENIA
Diarrhoea	5	BACTERIAL DIARRHOEA; DIARRHOEA; DIARRHOEA HAEMORRHAGIC; DIARRHOEA INFECTIOUS; VIRAL DIARRHOEA
Hypertension	14	BLOOD PRESSURE INCREASED; ESSENTIAL HYPERTENSION; HYPERTENSION; HYPERTENSIVE CRISIS; IDIOPATHIC INTRACRANIAL HYPERTENSION; LABILE HYPERTENSION; MALIGNANT HYPERTENSION; ORTHOSTATIC HYPERTENSION; PORTAL HYPERTENSION; PROCEDURAL HYPERTENSION; PULMONARY ARTERIAL HYPERTENSION; PULMONARY HYPERTENSION; PULMONARY VENOUS HYPERTENSION; SYSTOLIC HYPERTENSION
Infection any	247	ABDOMINAL INFECTION; ABDOMINAL SEPSIS; ACANTHAMOEBA INFECTION; ACINETOBACTER BACTERAEMIA; ACINETOBACTER INFECTION; ADENOVIRUS INFECTION; ALPHA HAEMOLYTIC STREPTOCOCCAL INFECTION; ASPERGILLOMA; ASPERGILLUS INFECTION; ASPERGILLUS TEST POSITIVE; ATYPICAL MYCOBACTERIAL INFECTION; ATYPICAL PNEUMONIA; BACILLUS BACTERAEMIA; BACTERAEMIA; BACTERIAL INFECTION; BACTERIAL SEPSIS; BILIARY SEPSIS; BRONCHOPULMONARY ASPERGILLOSIS; CAMPYLOBACTER INFECTION; CANDIDA INFECTION; CANDIDA PNEUMONIA; CATHETER SITE INFECTION; CENTRAL NERVOUS SYSTEM FUNGAL

		INFECTION; CENTRAL NERVOUS SYSTEM INFECTION; CEREBRAL ASPERGILLOSIS; CEREBRAL FUNGAL INFECTION; CLOSTRIDIAL INFECTION; CLOSTRIDIAL SEPSIS; CLOSTRIDIUM BACTERAEMIA; CLOSTRIDIUM DIFFICILE INFECTION; CORONAVIRUS INFECTION; COVID-19; COVID-19 IMMUNISATION; COVID-19 PNEUMONIA; CRYPTOCOCCAL CUTANEOUS INFECTION; CRYPTOSPORIDIOSIS INFECTION; CYTOMEGALOVIRUS GASTROINTESTINAL INFECTION; CYTOMEGALOVIRUS INFECTION; CYTOMEGALOVIRUS INFECTION REACTIVATION; DEVICE RELATED INFECTION; DEVICE RELATED SEPSIS; DISSEMINATED ASPERGILLOSIS; DISSEMINATED VARICELLA ZOSTER VIRUS INFECTION; EAR INFECTION; EAR INFECTION STAPHYLOCOCCAL; EAR INFECTION VIRAL; ENCEPHALITIS FUNGAL; ENTEROBACTER INFECTION; ENTEROBACTER SEPSIS; ENTEROCOCCAL BACTERAEMIA; ENTEROCOCCAL INFECTION; ENTEROCOCCAL SEPSIS; ENTEROVIRUS INFECTION; EOSINOPHILIC PNEUMONIA; EPSTEIN-BARR VIRUS INFECTION; EPSTEIN-BARR VIRUS INFECTION REACTIVATION; ESCHERICHIA BACTERAEMIA; ESCHERICHIA INFECTION; ESCHERICHIA SEPSIS; ESCHERICHIA URINARY TRACT INFECTION; EYE INFECTION; EYE INFECTION BACTERIAL; EYE INFECTION FUNGAL; EYE INFECTION STAPHYLOCOCCAL; EYE INFECTION VIRAL; EYELID INFECTION; FEBRILE INFECTION; FLAVIVIRUS INFECTION; FUNGAL ABSCESS CENTRAL NERVOUS SYSTEM; FUNGAL CYSTITIS; FUNGAL DISEASE CARRIER; FUNGAL FOOT INFECTION; FUNGAL INFECTION; FUNGAL OESOPHAGITIS; FUNGAL SEPSIS; FUNGAL SKIN INFECTION; FUNGAL TEST POSITIVE; FUSARIUM INFECTION; GASTRIC INFECTION; GASTROINTESTINAL BACTERIAL INFECTION; GASTROINTESTINAL INFECTION; GASTROINTESTINAL VIRAL INFECTION; GENITAL HERPES ZOSTER; GENITOURINARY TRACT INFECTION; GROIN INFECTION; HAEMATOMA INFECTION; HAEMOPHILUS INFECTION; HAEMORRHAGIC PNEUMONIA; HELICOBACTER INFECTION; HELMINTHIC INFECTION; HEPATIC INFECTION; HERPES VIRUS INFECTION; HERPES ZOSTER; HERPES ZOSTER OTICUS; HERPES ZOSTER REACTIVATION; HUMAN HERPESVIRUS 6 INFECTION; INFECTION; INFECTION IN AN IMMUNOCOMPROMISED HOST; INFECTION SUSCEPTIBILITY INCREASED; INFUSION SITE INFECTION; INJECTION SITE INFECTION; JC VIRUS INFECTION; KIDNEY INFECTION; KLEBSIELLA BACTERAEMIA; KLEBSIELLA INFECTION; KLEBSIELLA SEPSIS; LARGE INTESTINE INFECTION; LEGIONELLA INFECTION; LISTERIA SEPSIS; LOCALISED INFECTION; LOWER RESPIRATORY TRACT INFECTION; LOWER RESPIRATORY TRACT INFECTION BACTERIAL; LOWER RESPIRATORY TRACT INFECTION FUNGAL; LOWER RESPIRATORY TRACT INFECTION VIRAL; LYMPH GLAND INFECTION; MEDICAL DEVICE SITE INFECTION; MENINGITIS ASEPTIC; MENINGITIS ASPERGILLUS; MENINGITIS FUNGAL; METAPNEUMOVIRUS INFECTION; METAPNEUMOVIRUS PNEUMONIA; MICROSPORIDIA INFECTION; MORGANELLA INFECTION; MYCOBACTERIAL INFECTION; MYCOBACTERIUM AVIUM COMPLEX INFECTION; MYCOBACTERIUM KANSASII INFECTION; NAIL BED INFECTION; NAIL INFECTION; NECROTISING FASCIITIS FUNGAL; NEUTROPENIC INFECTION; NEUTROPENIC SEPSIS; NOSOCOMIAL INFECTION; OPHTHALMIC HERPES ZOSTER; OPPORTUNISTIC INFECTION; ORAL BACTERIAL INFECTION; ORAL FUNGAL INFECTION; ORAL HERPES ZOSTER; ORAL INFECTION; ORAL VIRAL INFECTION; ORGANISING PNEUMONIA; OSTEOMYELITIS FUNGAL; PANCREAS INFECTION; PAPILLOMA VIRAL INFECTION; PARAINFLUENZAE VIRUS INFECTION; PARECHOVIRUS INFECTION; PENILE INFECTION; PERINEAL INFECTION; PERIORBITAL INFECTION; PLEURAL INFECTION; PLEURAL INFECTION BACTERIAL; PNEUMOCOCCAL SEPSIS; PNEUMOCYSTIS JIROVECII INFECTION; PNEUMOCYSTIS JIROVECII PNEUMONIA; PNEUMONIA; PNEUMONIA ACINETOBACTER; PNEUMONIA

		ASPIRATION; PNEUMONIA BACTERIAL; PNEUMONIA CRYPTOCOCCAL; PNEUMONIA CYTOMEGALOVIRAL; PNEUMONIA ESCHERICHIA; PNEUMONIA FUNGAL; PNEUMONIA HAEMOPHILUS; PNEUMONIA INFLUENZAL; PNEUMONIA KLEBSIELLA; PNEUMONIA LEGIONELLA; PNEUMONIA MORAXELLA; PNEUMONIA MYCOPLASMAL; PNEUMONIA NECROTISING; PNEUMONIA PNEUMOCOCCAL; PNEUMONIA PSEUDOMONAL; PNEUMONIA RESPIRATORY SYNCYTIAL VIRAL; PNEUMONIA SERRATIA; PNEUMONIA STAPHYLOCOCCAL; PNEUMONIA STREPTOCOCCAL; PNEUMONIA VIRAL; POST PROCEDURAL INFECTION; POST PROCEDURAL PNEUMONIA; POST PROCEDURAL SEPSIS; POST-ACUTE COVID-19 SYNDROME; POSTOPERATIVE WOUND INFECTION; PROSTATE INFECTION; PROTEUS INFECTION; PSEUDOMONAL BACTERAEMIA; PSEUDOMONAL SEPSIS; PSEUDOMONAS INFECTION; PULMONARY SEPSIS; RESPIRATORY SYNCYTIAL VIRUS INFECTION; RESPIRATORY TRACT INFECTION; RESPIRATORY TRACT INFECTION BACTERIAL; RESPIRATORY TRACT INFECTION FUNGAL; RESPIRATORY TRACT INFECTION VIRAL; RHINOVIRUS INFECTION; ROTAVIRUS INFECTION; SALMONELLA SEPSIS; SCROTAL INFECTION; SEPSIS; SEPSIS SYNDROME; SEPTIC PULMONARY EMBOLISM; SEPTIC RASH; SEPTIC SHOCK; SERRATIA INFECTION; SHIGELLA INFECTION; SINUSITIS FUNGAL; SKIN BACTERIAL INFECTION; SKIN INFECTION; SOFT TISSUE INFECTION; SPLENIC INFECTION; STAPHYLOCOCCAL BACTERAEMIA; STAPHYLOCOCCAL INFECTION; STAPHYLOCOCCAL SEPSIS; STAPHYLOCOCCAL SKIN INFECTION; STOMA SITE INFECTION; STOMATOCOCCAL INFECTION; STREPTOCOCCAL BACTERAEMIA; STREPTOCOCCAL INFECTION; STREPTOCOCCAL SEPSIS; STREPTOCOCCAL URINARY TRACT INFECTION; SUPERINFECTION; SUPERINFECTION FUNGAL; SUSPECTED COVID-19; SYSTEMIC CANDIDA; SYSTEMIC INFECTION; SYSTEMIC VIRAL INFECTION; TONGUE FUNGAL INFECTION; TOOTH INFECTION; UPPER RESPIRATORY FUNGAL INFECTION; UPPER RESPIRATORY TRACT INFECTION; URINARY TRACT INFECTION; URINARY TRACT INFECTION BACTERIAL; URINARY TRACT INFECTION ENTEROCOCCAL; URINARY TRACT INFECTION FUNGAL; URINARY TRACT INFECTION PSEUDOMONAL; URINARY TRACT INFECTION STAPHYLOCOCCAL; UROSEPSIS; VAGINAL INFECTION; VARICELLA ZOSTER PNEUMONIA; VARICELLA ZOSTER VIRUS INFECTION; VIRAL INFECTION; VIRAL SKIN INFECTION; VIRAL UPPER RESPIRATORY TRACT INFECTION; VULVOVAGINAL HUMAN PAPILLOMA VIRUS INFECTION; VULVOVAGINAL MYCOTIC INFECTION; WEST NILE VIRAL INFECTION; WOUND INFECTION; WOUND INFECTION STAPHYLOCOCCAL; WOUND SEPSIS; YERSINIA INFECTION
Major bleeding like	7	CEREBRAL HAEMORRHAGE; GASTROINTESTINAL HAEMORRHAGE; HAEMORRHAGIC STROKE; LOWER GASTROINTESTINAL HAEMORRHAGE; SUBDURAL HAEMATOMA; TRAUMATIC INTRACRANIAL HAEMORRHAGE; UPPER GASTROINTESTINAL HAEMORRHAGE
Severe infection like	80	ABDOMINAL SEPSIS; ACINETOBACTER BACTERAEMIA; ASPERGILLOMA; ASPERGILLUS INFECTION; ASPERGILLUS TEST POSITIVE; ATYPICAL PNEUMONIA; BACILLUS BACTERAEMIA; BACTERAEMIA; BACTERIAL SEPSIS; BILIARY SEPSIS; BRONCHOPULMONARY ASPERGILLOSIS; CANDIDA PNEUMONIA; CENTRAL NERVOUS SYSTEM FUNGAL INFECTION; CEREBRAL ASPERGILLOSIS; CEREBRAL FUNGAL INFECTION; CLOSTRIDIAL SEPSIS; CLOSTRIDIUM BACTERAEMIA; COVID-19 PNEUMONIA; DEVICE RELATED SEPSIS; DISSEMINATED ASPERGILLOSIS; ENTEROBACTER SEPSIS; ENTEROCOCCAL BACTERAEMIA; ENTEROCOCCAL SEPSIS; EOSINOPHILIC PNEUMONIA; ESCHERICHIA BACTERAEMIA; ESCHERICHIA SEPSIS; FUNGAL INFECTION; FUNGAL SEPSIS; HAEMORRHAGIC PNEUMONIA; KLEBSIELLA BACTERAEMIA; KLEBSIELLA SEPSIS; LISTERIA SEPSIS; MENINGITIS ASPERGILLUS;

		METAPNEUMOVIRUS PNEUMONIA; NEUTROPENIC SEPSIS; OPPORTUNISTIC INFECTION; ORAL FUNGAL INFECTION; ORGANISING PNEUMONIA; PNEUMOCOCCAL SEPSIS; PNEUMOCYSTIS JIROVECII PNEUMONIA; PNEUMONIA; PNEUMONIA ACINETOBACTER; PNEUMONIA ASPIRATION; PNEUMONIA BACTERIAL; PNEUMONIA CRYPTOCOCCAL; PNEUMONIA CYTOMEGALOVIRAL; PNEUMONIA ESCHERICHIA; PNEUMONIA FUNGAL; PNEUMONIA HAEMOPHILUS; PNEUMONIA INFLUENZAL; PNEUMONIA KLEBSIELLA; PNEUMONIA LEGIONELLA; PNEUMONIA MORAXELLA; PNEUMONIA MYCOPLASMAL; PNEUMONIA NECROTISING; PNEUMONIA PNEUMOCOCCAL; PNEUMONIA PSEUDOMONAL; PNEUMONIA RESPIRATORY SYNCYTIAL VIRAL; PNEUMONIA SERRATIA; PNEUMONIA STAPHYLOCOCCAL; PNEUMONIA STREPTOCOCCAL; PNEUMONIA VIRAL; POST PROCEDURAL PNEUMONIA; POST PROCEDURAL SEPSIS; PSEUDOMONAL BACTERAEMIA; PSEUDOMONAL SEPSIS; PULMONARY SEPSIS; SALMONELLA SEPSIS; SEPSIS; SEPSIS SYNDROME; SEPTIC SHOCK; STAPHYLOCOCCAL BACTERAEMIA; STAPHYLOCOCCAL SEPSIS; STREPTOCOCCAL BACTERAEMIA; STREPTOCOCCAL SEPSIS; TONGUE FUNGAL INFECTION; UPPER RESPIRATORY FUNGAL INFECTION; UROSEPSIS; VARICELLA ZOSTER PNEUMONIA; WOUND SEPSIS

Supplementary Table S3. Final analysis summary.

Item	Value
FAERS version	25Q2
Cohort linking mode	report level
Adult CLL SLL ibrutinib cohort n	20878
CLL reports in cohort n	19999
SLL reports in cohort n	705
CLL plus SLL reports in cohort n	174
Excluded false positive indication rows n	230
CYP3A modifier n	1373
CYP3A inhibitor n	1075
CYP3A strong inhibitor n	242
CYP3A moderate inhibitor n	862
CYP3A inducer n	367
Antithrombotic n	3701
Anticoagulant n	1670
Antiplatelet n	2382
Allowed comed roles	PS, SS, C, I
min pt total	3

Supplementary Table S4. Cohort distribution by diagnosis, age group and sex.

Diagnosis group	Age group	Sex	N
CLL	18-64	Female	1810
CLL	18-64	Male	3543
CLL	18-64	Unknown	58
CLL	65-74	Female	2503
CLL	65-74	Male	4461
CLL	65-74	Unknown	52
CLL	75+	Female	3148
CLL	75+	Male	4353
CLL	75+	Unknown	71
CLL+SLL	18-64	Female	15
CLL+SLL	18-64	Male	32
CLL+SLL	65-74	Female	25
CLL+SLL	65-74	Male	42
CLL+SLL	75+	Female	25
CLL+SLL	75+	Male	34
CLL+SLL	75+	Unknown	1
SLL	18-64	Female	51
SLL	18-64	Male	110
SLL	18-64	Unknown	2
SLL	65-74	Female	95
SLL	65-74	Male	158
SLL	65-74	Unknown	1
SLL	75+	Female	107
SLL	75+	Male	180

SLL	75+	Unknown	1

Supplementary Table S5. Overall co-exposure summary.

Exposure	Exposed reports	Comparator reports
Any CYP3A modifier	1,373 (6.6%)	19,505 (93.4%)
CYP3A inhibitor	1,075 (5.1%)	19,803 (94.9%)
Strong CYP3A inhibitor	242 (1.2%)	20,636 (98.8%)
Moderate CYP3A inhibitor	862 (4.1%)	20,016 (95.9%)
CYP3A inducer	367 (1.8%)	20,511 (98.2%)
Any antithrombotic	3,701 (17.7%)	17,177 (82.3%)
Anticoagulant	1,670 (8.0%)	19,208 (92.0%)
Antiplatelet	2,382 (11.4%)	18,496 (88.6%)

Supplementary Table S6. Co-exposure summary by age group.

Stratum	N	Any CYP3A modifier n (%)	CYP3A inhibitor n (%)	CYP3A inducer n (%)	Any antithrombotic n (%)	Anticoagulant n (%)	Antiplatelet n (%)
18-64	5621	362 (6.4%)	271 (4.8%)	133 (2.4%)	551 (9.8%)	231 (4.1%)	352 (6.3%)
65-74	7337	531 (7.2%)	406 (5.5%)	141 (1.9%)	1,310 (17.9%)	579 (7.9%)	860 (11.7%)
75+	7920	480 (6.1%)	398 (5.0%)	93 (1.2%)	1,840 (23.2%)	860 (10.9%)	1,170 (14.8%)

Supplementary Table S7. Co-exposure summary by sex.

Stratum	N	Any CYP3A modifier n (%)	CYP3A inhibitor n (%)	CYP3A inducer n (%)	Any antithrombotic n (%)	Anticoagulant n (%)	Antiplatelet n (%)
Female	7779	452 (5.8%)	364 (4.7%)	103 (1.3%)	1,259 (16.2%)	546 (7.0%)	828 (10.6%)
Male	12913	907 (7.0%)	703 (5.4%)	258 (2.0%)	2,423 (18.8%)	1,112 (8.6%)	1,544 (12.0%)
Unknown	186	14 (7.5%)	8 (4.3%)	6 (3.2%)	19 (10.2%)	12 (6.5%)	10 (5.4%)

Supplementary Table S8. Overall AE-group signal results for all co-exposure analyses.

Exposure analysis	AE group	a	c	ROR	ROR025	ROR975	PRR	Chi-square	IC	IC025	IC975	Positive algorithms
Any CYP3A modifier	Severe infection like	260	1961	2.09	1.81	2.41	1.88	106.47	0.83	0.68	0.98	2
Any CYP3A modifier	Cytopenia	168	1247	2.04	1.72	2.42	1.91	69.31	0.85	0.66	1.04	2
Any CYP3A modifier	Infection any	432	3795	1.90	1.69	2.14	1.62	114.53	0.64	0.53	0.74	2
Any CYP3A modifier	Atrial arrhythmia	144	1134	1.90	1.58	2.28	1.80	48.77	0.78	0.57	0.99	2
Any CYP3A modifier	Major bleeding like	52	486	1.54	1.15	2.06	1.52	8.58	0.56	0.19	0.92	2
Any CYP3A modifier	Bleeding any	255	2844	1.34	1.16	1.54	1.27	16.17	0.32	0.17	0.48	2
Any CYP3A modifier	Hypertension	58	696	1.19	0.91	1.57	1.18	1.59	0.23	-0.12	0.58	0
Any CYP3A modifier	Diarrhoea	102	1371	1.06	0.86	1.31	1.06	0.31	0.07	-0.19	0.33	0
CYP3A inhibitor	Atrial arrhythmia	124	1154	2.11	1.73	2.56	1.98	57.80	0.91	0.69	1.14	2
CYP3A inhibitor	Severe infection like	202	2019	2.04	1.74	2.39	1.84	79.24	0.82	0.65	0.99	2
CYP3A inhibitor	Infection any	336	3891	1.86	1.63	2.13	1.59	85.08	0.63	0.50	0.75	2
CYP3A inhibitor	Cytopenia	121	1294	1.81	1.49	2.21	1.72	35.98	0.73	0.50	0.96	2
CYP3A inhibitor	Major bleeding like	42	496	1.58	1.15	2.18	1.56	7.99	0.60	0.19	1.01	2
CYP3A inhibitor	Bleeding any	213	2886	1.45	1.24	1.69	1.36	22.15	0.42	0.25	0.58	2
CYP3A inhibitor	Hypertension	49	705	1.29	0.96	1.74	1.28	2.92	0.34	-0.05	0.72	0
CYP3A inhibitor	Diarrhoea	89	1384	1.20	0.96	1.50	1.18	2.59	0.23	-0.05	0.51	0
Strong CYP3A inhibitor	Severe infection like	91	2130	5.24	4.02	6.82	3.64	187.27	1.82	1.59	2.05	3
Strong CYP3A inhibitor	Infection any	124	4103	4.23	3.28	5.46	2.58	145.65	1.34	1.17	1.51	3
Strong CYP3A inhibitor	Cytopenia	45	1370	3.21	2.31	4.46	2.80	54.12	1.46	1.08	1.83	3
Strong CYP3A inhibitor	Bleeding any	50	3049	1.50	1.10	2.06	1.40	6.56	0.48	0.12	0.83	2
Strong CYP3A inhibitor	Diarrhoea	11	1462	0.62	0.34	1.15	0.64	2.35	-0.63	-1.46	0.20	0
Strong CYP3A inhibitor	Atrial arrhythmia	8	1270	0.52	0.26	1.06	0.54	3.38	-0.89	-1.87	0.09	0
Strong CYP3A inhibitor	Major bleeding like	3	535	0.47	0.15	1.48	0.48	1.74	-1.06	-2.67	0.56	0

Strong CYP3A inhibitor	Hypertension	3	751	0.33	0.11	1.04	0.34	3.96	-1.54	-3.16	0.08	0
Moderate CYP3A inhibitor	Atrial arrhythmia	118	1160	2.58	2.10	3.16	2.36	89.61	1.16	0.93	1.39	3
Moderate CYP3A inhibitor	Major bleeding like	40	498	1.91	1.37	2.65	1.87	15.25	0.85	0.43	1.27	2
Moderate CYP3A inhibitor	Hypertension	46	708	1.54	1.13	2.09	1.51	7.69	0.56	0.17	0.96	2
Moderate CYP3A inhibitor	Bleeding any	171	2928	1.44	1.22	1.72	1.36	17.74	0.42	0.23	0.61	2
Moderate CYP3A inhibitor	Infection any	228	3999	1.44	1.23	1.68	1.32	21.43	0.39	0.23	0.54	2
Moderate CYP3A inhibitor	Cytopenia	79	1336	1.41	1.11	1.79	1.37	8.11	0.44	0.14	0.73	2
Moderate CYP3A inhibitor	Severe infection like	120	2101	1.38	1.13	1.68	1.33	10.19	0.39	0.16	0.62	2
Moderate CYP3A inhibitor	Diarrhoea	80	1393	1.37	1.08	1.73	1.33	6.79	0.40	0.10	0.69	2
CYP3A inducer	Cytopenia	71	1344	3.42	2.62	4.46	2.95	93.40	1.51	1.22	1.81	3
CYP3A inducer	Severe infection like	82	2139	2.47	1.93	3.17	2.14	53.84	1.07	0.80	1.34	3
CYP3A inducer	Infection any	131	4096	2.22	1.79	2.76	1.79	55.21	0.82	0.62	1.01	2
CYP3A inducer	Major bleeding like	10	528	1.06	0.56	2.00	1.06	0.03	0.08	-0.79	0.95	0
CYP3A inducer	Atrial arrhythmia	23	1255	1.03	0.67	1.57	1.02	0.01	0.03	-0.53	0.60	0
CYP3A inducer	Bleeding any	49	3050	0.88	0.65	1.19	0.90	0.66	-0.15	-0.53	0.22	0
CYP3A inducer	Hypertension	11	743	0.82	0.45	1.50	0.83	0.40	-0.27	-1.10	0.56	0
CYP3A inducer	Diarrhoea	17	1456	0.64	0.39	1.04	0.65	3.34	-0.61	-1.27	0.06	0
Any antithrombotic	Atrial arrhythmia	438	840	2.61	2.31	2.95	2.42	255.53	0.95	0.85	1.05	3
Any antithrombotic	Major bleeding like	182	356	2.44	2.04	2.93	2.37	98.18	0.93	0.77	1.10	3
Any antithrombotic	Bleeding any	902	2197	2.20	2.01	2.40	1.91	323.11	0.72	0.65	0.78	2
Any antithrombotic	Hypertension	167	587	1.34	1.12	1.59	1.32	10.49	0.32	0.13	0.51	2
Any antithrombotic	Severe infection like	478	1743	1.31	1.18	1.46	1.27	24.54	0.28	0.17	0.39	2
Any antithrombotic	Diarrhoea	313	1160	1.28	1.12	1.45	1.25	13.48	0.26	0.13	0.40	2
Any antithrombotic	Infection any	811	3416	1.13	1.04	1.23	1.10	7.74	0.11	0.04	0.19	2
Any antithrombotic	Cytopenia	276	1139	1.13	0.99	1.30	1.12	3.29	0.14	-0.01	0.28	0
Anticoagulant	Atrial arrhythmia	302	976	4.12	3.58	4.74	3.56	452.02	1.56	1.44	1.69	3
Anticoagulant	Bleeding any	533	2566	3.04	2.72	3.40	2.39	418.58	1.10	1.01	1.19	3

Anticoagulant	Major bleeding like	107	431	2.98	2.40	3.71	2.86	106.08	1.31	1.08	1.55	3
Anticoagulant	Severe infection like	226	1995	1.35	1.16	1.57	1.30	16.00	0.35	0.18	0.51	2
Anticoagulant	Cytopenia	134	1281	1.22	1.01	1.47	1.20	4.46	0.24	0.02	0.47	2
Anticoagulant	Infection any	389	3838	1.22	1.08	1.37	1.17	10.44	0.20	0.08	0.32	2
Anticoagulant	Hypertension	70	684	1.18	0.92	1.52	1.18	1.76	0.21	-0.10	0.53	0
Anticoagulant	Diarrhoea	127	1346	1.09	0.90	1.32	1.09	0.84	0.11	-0.12	0.34	0
Antiplatelet	Major bleeding like	102	436	1.85	1.49	2.31	1.82	31.14	0.73	0.49	0.98	2
Antiplatelet	Bleeding any	487	2612	1.56	1.40	1.74	1.45	66.75	0.46	0.36	0.57	2
Antiplatelet	Atrial arrhythmia	206	1072	1.54	1.32	1.80	1.49	29.88	0.50	0.33	0.67	2
Antiplatelet	Hypertension	113	641	1.39	1.13	1.70	1.37	9.91	0.39	0.15	0.63	2
Antiplatelet	Diarrhoea	217	1256	1.38	1.18	1.60	1.34	17.31	0.37	0.20	0.54	2
Antiplatelet	Severe infection like	312	1909	1.31	1.15	1.49	1.27	17.12	0.30	0.16	0.44	2
Antiplatelet	Infection any	508	3719	1.08	0.97	1.20	1.06	1.94	0.08	-0.03	0.18	0
Antiplatelet	Cytopenia	164	1251	1.02	0.86	1.21	1.02	0.05	0.02	-0.18	0.22	0

Supplementary Table S9. Age-stratified AE-group signal results.

Exposure analysis	Age group	AE group	a	c	ROR (95% CI)	Positive algorithms
Any CYP3A modifier	18-64	Severe infection like	97	508	3.42 (2.67–4.40)	3
Any CYP3A modifier	18-64	Cytopenia	56	387	2.30 (1.70–3.12)	3
Any CYP3A modifier	18-64	Infection any	141	1059	2.53 (2.03–3.16)	2
Any CYP3A modifier	18-64	Bleeding any	60	662	1.38 (1.03–1.84)	2
Any CYP3A modifier	18-64	Major bleeding like	9	83	1.59 (0.79–3.19)	0
Any CYP3A modifier	18-64	Hypertension	14	155	1.32 (0.76–2.31)	0
Any CYP3A modifier	18-64	Atrial arrhythmia	21	236	1.31 (0.83–2.08)	0
Any CYP3A modifier	18-64	Diarrhoea	28	354	1.16 (0.78–1.73)	0
Any CYP3A modifier	65-74	Cytopenia	72	484	2.05 (1.57–2.67)	2
Any CYP3A modifier	65-74	Infection any	181	1382	2.03 (1.68–2.45)	2
Any CYP3A modifier	65-74	Atrial arrhythmia	66	448	2.01 (1.53–2.65)	2
Any CYP3A modifier	65-74	Severe infection like	101	715	2.00 (1.59–2.52)	2
Any CYP3A modifier	65-74	Major bleeding like	22	179	1.60 (1.02–2.51)	2
Any CYP3A modifier	65-74	Bleeding any	104	1036	1.36 (1.08–1.70)	2
Any CYP3A modifier	65-74	Diarrhoea	42	473	1.15 (0.83–1.60)	0
Any CYP3A modifier	65-74	Hypertension	22	292	0.96 (0.62–1.50)	0
Any CYP3A modifier	75+	Atrial arrhythmia	57	450	2.09 (1.56–2.80)	2
Any CYP3A modifier	75+	Cytopenia	40	376	1.71 (1.22–2.40)	2
Any CYP3A modifier	75+	Severe infection like	62	738	1.35 (1.02–1.78)	2
Any CYP3A modifier	75+	Infection any	110	1354	1.34 (1.07–1.67)	2
Any CYP3A modifier	75+	Bleeding any	91	1146	1.28 (1.01–1.63)	2
Any CYP3A modifier	75+	Major bleeding like	21	224	1.47 (0.93–2.33)	0
Any CYP3A modifier	75+	Hypertension	22	249	1.39 (0.89–2.17)	0
Any CYP3A modifier	75+	Diarrhoea	32	544	0.91 (0.63–1.31)	0
CYP3A inhibitor	18-64	Severe infection like	75	530	3.48 (2.63–4.61)	3
CYP3A inhibitor	18-64	Infection any	112	1088	2.76 (2.15–3.55)	3
CYP3A inhibitor	18-64	Cytopenia	43	400	2.33 (1.66–3.28)	3
CYP3A inhibitor	18-64	Bleeding any	52	670	1.66 (1.21–2.27)	2
CYP3A inhibitor	18-64	Atrial arrhythmia	19	238	1.62 (1.00–2.63)	1
CYP3A inhibitor	18-64	Hypertension	12	157	1.53 (0.84–2.79)	0
CYP3A inhibitor	18-64	Diarrhoea	25	357	1.42 (0.93–2.17)	0
CYP3A inhibitor	18-64	Major bleeding like	5	87	1.14 (0.46–2.82)	0
CYP3A inhibitor	65-74	Atrial arrhythmia	53	461	2.11 (1.56–2.86)	2

CYP3A inhibitor	65-74	Infection any	132	1431	1.85 (1.49–2.30)	2
CYP3A inhibitor	65-74	Cytopenia	51	505	1.83 (1.34–2.49)	2
CYP3A inhibitor	65-74	Severe infection like	73	743	1.83 (1.40–2.38)	2
CYP3A inhibitor	65-74	Major bleeding like	18	183	1.71 (1.04–2.81)	2
CYP3A inhibitor	65-74	Bleeding any	79	1061	1.34 (1.04–1.72)	2
CYP3A inhibitor	65-74	Diarrhoea	37	478	1.35 (0.95–1.92)	0
CYP3A inhibitor	65-74	Hypertension	20	294	1.17 (0.74–1.86)	0
CYP3A inhibitor	75+	Atrial arrhythmia	52	455	2.33 (1.72–3.17)	3
CYP3A inhibitor	75+	Major bleeding like	19	226	1.62 (1.00–2.61)	2
CYP3A inhibitor	75+	Bleeding any	82	1155	1.43 (1.11–1.84)	2
CYP3A inhibitor	75+	Severe infection like	54	746	1.43 (1.06–1.92)	2
CYP3A inhibitor	75+	Infection any	92	1372	1.35 (1.06–1.71)	2
CYP3A inhibitor	75+	Cytopenia	27	389	1.33 (0.89–2.00)	0
CYP3A inhibitor	75+	Hypertension	17	254	1.28 (0.77–2.11)	0
CYP3A inhibitor	75+	Diarrhoea	27	549	0.92 (0.62–1.38)	0
CYP3A inducer	18-64	Cytopenia	35	408	4.45 (2.98–6.63)	3
CYP3A inducer	18-64	Severe infection like	42	563	4.04 (2.77–5.88)	3
CYP3A inducer	18-64	Infection any	57	1143	2.85 (2.01–4.04)	3
CYP3A inducer	18-64	Major bleeding like	4	88	1.90 (0.69–5.26)	0
CYP3A inducer	18-64	Bleeding any	14	708	0.79 (0.45–1.39)	0
CYP3A inducer	18-64	Diarrhoea	7	375	0.76 (0.35–1.63)	0
CYP3A inducer	18-64	Hypertension	2	167	0.49 (0.12–1.98)	0
CYP3A inducer	18-64	Atrial arrhythmia	2	255	0.31 (0.08–1.27)	0
CYP3A inducer	65-74	Cytopenia	23	533	2.44 (1.55–3.84)	3
CYP3A inducer	65-74	Infection any	53	1510	2.27 (1.61–3.20)	2
CYP3A inducer	65-74	Severe infection like	29	787	2.11 (1.39–3.19)	2
CYP3A inducer	65-74	Atrial arrhythmia	14	500	1.48 (0.84–2.58)	0
CYP3A inducer	65-74	Bleeding any	26	1114	1.23 (0.80–1.90)	0
CYP3A inducer	65-74	Major bleeding like	4	197	1.04 (0.38–2.83)	0
CYP3A inducer	65-74	Diarrhoea	5	510	0.48 (0.20–1.18)	0
CYP3A inducer	65-74	Hypertension	3	311	0.48 (0.15–1.52)	0
CYP3A inducer	75+	Cytopenia	13	403	2.99 (1.65–5.43)	3
CYP3A inducer	75+	Hypertension	6	265	1.97 (0.85–4.54)	0
CYP3A inducer	75+	Infection any	21	1443	1.29 (0.79–2.11)	0
CYP3A inducer	75+	Severe infection like	11	789	1.20 (0.63–2.25)	0
CYP3A inducer	75+	Atrial arrhythmia	7	500	1.19 (0.55–2.59)	0

CYP3A inducer	75+	Diarrhoea	5	571	0.72 (0.29–1.78)	0
CYP3A inducer	75+	Major bleeding like	2	243	0.69 (0.17–2.80)	0
CYP3A inducer	75+	Bleeding any	9	1228	0.58 (0.29–1.15)	0
Any antithrombotic	18-64	Atrial arrhythmia	54	203	2.60 (1.90–3.57)	3
Any antithrombotic	18-64	Bleeding any	114	608	1.91 (1.53–2.39)	2
Any antithrombotic	18-64	Diarrhoea	58	324	1.72 (1.28–2.31)	2
Any antithrombotic	18-64	Hypertension	26	143	1.71 (1.11–2.62)	2
Any antithrombotic	18-64	Major bleeding like	12	80	1.39 (0.75–2.56)	0
Any antithrombotic	18-64	Severe infection like	65	540	1.12 (0.85–1.48)	0
Any antithrombotic	18-64	Infection any	126	1074	1.10 (0.89–1.36)	0
Any antithrombotic	18-64	Cytopenia	42	401	0.96 (0.69–1.34)	0
Any antithrombotic	65-74	Atrial arrhythmia	181	333	2.74 (2.26–3.32)	3
Any antithrombotic	65-74	Major bleeding like	72	129	2.66 (1.98–3.57)	3
Any antithrombotic	65-74	Bleeding any	337	803	2.25 (1.95–2.60)	2
Any antithrombotic	65-74	Hypertension	75	239	1.47 (1.13–1.92)	2
Any antithrombotic	65-74	Severe infection like	176	640	1.31 (1.09–1.56)	2
Any antithrombotic	65-74	Infection any	318	1245	1.23 (1.07–1.42)	2
Any antithrombotic	65-74	Diarrhoea	103	412	1.16 (0.93–1.46)	0
Any antithrombotic	65-74	Cytopenia	106	450	1.09 (0.88–1.36)	0
Any antithrombotic	75+	Atrial arrhythmia	203	304	2.36 (1.96–2.84)	3
Any antithrombotic	75+	Major bleeding like	98	147	2.27 (1.75–2.95)	3
Any antithrombotic	75+	Bleeding any	451	786	2.19 (1.92–2.49)	2
Any antithrombotic	75+	Cytopenia	128	288	1.50 (1.21–1.86)	2
Any antithrombotic	75+	Severe infection like	237	563	1.45 (1.23–1.70)	2
Any antithrombotic	75+	Diarrhoea	152	424	1.20 (0.99–1.46)	0
Any antithrombotic	75+	Infection any	367	1097	1.13 (0.99–1.29)	0
Any antithrombotic	75+	Hypertension	66	205	1.07 (0.80–1.41)	0
Anticoagulant	18-64	Atrial arrhythmia	32	225	3.69 (2.48–5.49)	3
Anticoagulant	18-64	Bleeding any	61	661	2.57 (1.89–3.48)	3
Anticoagulant	18-64	Cytopenia	24	419	1.38 (0.89–2.12)	0
Anticoagulant	18-64	Major bleeding like	5	87	1.35 (0.54–3.35)	0
Anticoagulant	18-64	Hypertension	9	160	1.33 (0.67–2.63)	0
Anticoagulant	18-64	Diarrhoea	20	362	1.32 (0.82–2.11)	0
Anticoagulant	18-64	Severe infection like	26	579	1.05 (0.69–1.60)	0
Anticoagulant	18-64	Infection any	48	1152	0.96 (0.70–1.33)	0
Anticoagulant	65-74	Atrial arrhythmia	133	381	4.99 (4.01–6.22)	3

Anticoagulant	65-74	Bleeding any	201	939	3.30 (2.74–3.96)	3
Anticoagulant	65-74	Major bleeding like	42	159	3.25 (2.28–4.61)	3
Anticoagulant	65-74	Severe infection like	91	725	1.55 (1.22–1.97)	2
Anticoagulant	65-74	Infection any	166	1397	1.54 (1.28–1.86)	2
Anticoagulant	65-74	Hypertension	35	279	1.49 (1.04–2.15)	2
Anticoagulant	65-74	Cytopenia	53	503	1.25 (0.93–1.69)	0
Anticoagulant	65-74	Diarrhoea	49	466	1.25 (0.92–1.70)	0
Anticoagulant	75+	Atrial arrhythmia	137	370	3.43 (2.78–4.23)	3
Anticoagulant	75+	Bleeding any	271	966	2.90 (2.48–3.40)	3
Anticoagulant	75+	Major bleeding like	60	185	2.79 (2.06–3.76)	3
Anticoagulant	75+	Severe infection like	109	691	1.34 (1.08–1.66)	2
Anticoagulant	75+	Cytopenia	57	359	1.32 (0.99–1.77)	0
Anticoagulant	75+	Infection any	175	1289	1.14 (0.96–1.36)	0
Anticoagulant	75+	Diarrhoea	58	518	0.91 (0.69–1.21)	0
Anticoagulant	75+	Hypertension	26	245	0.87 (0.58–1.31)	0
Antiplatelet	18-64	Atrial arrhythmia	28	229	1.90 (1.26–2.86)	2
Antiplatelet	18-64	Hypertension	18	151	1.83 (1.11–3.01)	2
Antiplatelet	18-64	Diarrhoea	39	343	1.79 (1.26–2.54)	2
Antiplatelet	18-64	Bleeding any	63	659	1.52 (1.15–2.03)	2
Antiplatelet	18-64	Severe infection like	46	559	1.27 (0.92–1.75)	0
Antiplatelet	18-64	Major bleeding like	7	85	1.24 (0.57–2.70)	0
Antiplatelet	18-64	Infection any	86	1114	1.21 (0.94–1.55)	0
Antiplatelet	18-64	Cytopenia	21	422	0.73 (0.46–1.15)	0
Antiplatelet	65-74	Major bleeding like	45	156	2.24 (1.59–3.14)	3
Antiplatelet	65-74	Bleeding any	188	952	1.62 (1.36–1.94)	2
Antiplatelet	65-74	Atrial arrhythmia	79	435	1.40 (1.09–1.81)	2
Antiplatelet	65-74	Hypertension	45	269	1.27 (0.92–1.76)	0
Antiplatelet	65-74	Severe infection like	104	712	1.11 (0.89–1.39)	0
Antiplatelet	65-74	Diarrhoea	64	451	1.07 (0.82–1.41)	0
Antiplatelet	65-74	Infection any	185	1378	1.01 (0.85–1.21)	0
Antiplatelet	65-74	Cytopenia	63	493	0.96 (0.73–1.26)	0
Antiplatelet	75+	Severe infection like	162	638	1.54 (1.28–1.85)	2
Antiplatelet	75+	Major bleeding like	50	195	1.50 (1.09–2.06)	2
Antiplatelet	75+	Diarrhoea	114	462	1.47 (1.18–1.82)	2
Antiplatelet	75+	Bleeding any	236	1001	1.45 (1.24–1.70)	2
Antiplatelet	75+	Atrial arrhythmia	99	408	1.44 (1.14–1.81)	2

Antiplatelet	75+	Cytopenia	80	336	1.40 (1.09–1.80)	2
Antiplatelet	75+	Hypertension	50	221	1.32 (0.96–1.80)	0
Antiplatelet	75+	Infection any	237	1227	1.14 (0.98–1.34)	0

Supplementary Table S10. Sex-stratified AE-group signal results.

Exposure analysis	Sex	AE group	a	c	ROR (95% CI)	Positive algorithms
Any CYP3A modifier	Male	Severe infection like	211	1332	2.43 (2.06–2.86)	3
Any CYP3A modifier	Male	Cytopenia	121	780	2.22 (1.81–2.72)	3
Any CYP3A modifier	Male	Infection any	317	2358	2.20 (1.90–2.54)	2
Any CYP3A modifier	Male	Atrial arrhythmia	96	777	1.71 (1.37–2.14)	2
Any CYP3A modifier	Male	Major bleeding like	31	329	1.26 (0.86–1.83)	0
Any CYP3A modifier	Male	Hypertension	35	392	1.19 (0.84–1.69)	0
Any CYP3A modifier	Male	Bleeding any	152	1796	1.14 (0.95–1.37)	0
Any CYP3A modifier	Male	Diarrhoea	59	742	1.06 (0.80–1.39)	0
Any CYP3A modifier	Female	Atrial arrhythmia	48	343	2.42 (1.76–3.33)	3
Any CYP3A modifier	Female	Major bleeding like	21	152	2.30 (1.44–3.67)	3
Any CYP3A modifier	Female	Bleeding any	103	1026	1.81 (1.44–2.28)	2
Any CYP3A modifier	Female	Cytopenia	46	454	1.72 (1.25–2.36)	2
Any CYP3A modifier	Female	Infection any	114	1418	1.41 (1.13–1.75)	2
Any CYP3A modifier	Female	Severe infection like	49	621	1.31 (0.97–1.79)	0
Any CYP3A modifier	Female	Hypertension	23	300	1.26 (0.81–1.94)	0
Any CYP3A modifier	Female	Diarrhoea	43	622	1.13 (0.82–1.57)	0
CYP3A inhibitor	Male	Severe infection like	166	1377	2.43 (2.03–2.92)	3
CYP3A inhibitor	Male	Infection any	248	2427	2.20 (1.87–2.58)	2
CYP3A inhibitor	Male	Cytopenia	86	815	1.95 (1.54–2.47)	2
CYP3A inhibitor	Male	Atrial arrhythmia	82	791	1.91 (1.50–2.43)	2
CYP3A inhibitor	Male	Bleeding any	128	1820	1.27 (1.04–1.55)	2
CYP3A inhibitor	Male	Hypertension	30	397	1.33 (0.91–1.94)	0
CYP3A inhibitor	Male	Major bleeding like	24	336	1.25 (0.82–1.90)	0
CYP3A inhibitor	Male	Diarrhoea	51	750	1.20 (0.89–1.60)	0
CYP3A inhibitor	Female	Atrial arrhythmia	42	349	2.64 (1.88–3.71)	3
CYP3A inhibitor	Female	Major bleeding like	18	155	2.44 (1.48–4.02)	3
CYP3A inhibitor	Female	Bleeding any	85	1044	1.86 (1.45–2.39)	2
CYP3A inhibitor	Female	Cytopenia	34	466	1.54 (1.07–2.21)	2
CYP3A inhibitor	Female	Infection any	87	1445	1.30 (1.01–1.66)	2
CYP3A inhibitor	Female	Hypertension	19	304	1.29 (0.80–2.07)	0
CYP3A inhibitor	Female	Diarrhoea	38	627	1.26 (0.89–1.78)	0
CYP3A inhibitor	Female	Severe infection like	36	634	1.17 (0.82–1.67)	0
CYP3A inducer	Male	Cytopenia	56	845	3.87 (2.86–5.25)	3

CYP3A inducer	Male	Severe infection like	67	1476	2.66 (2.00–3.53)	3
CYP3A inducer	Male	Infection any	101	2574	2.52 (1.95–3.25)	2
CYP3A inducer	Male	Atrial arrhythmia	17	856	0.97 (0.59–1.60)	0
CYP3A inducer	Male	Major bleeding like	7	353	0.97 (0.46–2.08)	0
CYP3A inducer	Male	Hypertension	7	420	0.81 (0.38–1.73)	0
CYP3A inducer	Male	Bleeding any	28	1920	0.68 (0.46–1.01)	0
CYP3A inducer	Male	Diarrhoea	11	790	0.67 (0.36–1.23)	0
CYP3A inducer	Female	Cytopenia	15	485	2.53 (1.45–4.40)	3
CYP3A inducer	Female	Severe infection like	15	655	1.83 (1.05–3.18)	2
CYP3A inducer	Female	Infection any	30	1502	1.69 (1.10–2.59)	2
CYP3A inducer	Female	Bleeding any	21	1108	1.52 (0.94–2.46)	0
CYP3A inducer	Female	Major bleeding like	3	170	1.32 (0.42–4.22)	0
CYP3A inducer	Female	Atrial arrhythmia	6	385	1.17 (0.51–2.69)	0
CYP3A inducer	Female	Hypertension	4	319	0.93 (0.34–2.55)	0
CYP3A inducer	Female	Diarrhoea	6	659	0.66 (0.29–1.51)	0
Any antithrombotic	Male	Major bleeding like	127	233	2.43 (1.95–3.04)	3
Any antithrombotic	Male	Atrial arrhythmia	297	576	2.40 (2.07–2.79)	3
Any antithrombotic	Male	Bleeding any	609	1339	2.29 (2.06–2.56)	2
Any antithrombotic	Male	Hypertension	109	318	1.51 (1.21–1.88)	2
Any antithrombotic	Male	Severe infection like	353	1190	1.33 (1.17–1.51)	2
Any antithrombotic	Male	Diarrhoea	180	621	1.28 (1.07–1.51)	2
Any antithrombotic	Male	Infection any	540	2135	1.12 (1.01–1.25)	2
Any antithrombotic	Male	Cytopenia	173	728	1.03 (0.87–1.22)	0
Any antithrombotic	Female	Atrial arrhythmia	140	251	3.12 (2.52–3.88)	3
Any antithrombotic	Female	Major bleeding like	55	118	2.48 (1.79–3.43)	3
Any antithrombotic	Female	Bleeding any	286	843	1.98 (1.70–2.30)	2
Any antithrombotic	Female	Cytopenia	102	398	1.36 (1.08–1.70)	2
Any antithrombotic	Female	Diarrhoea	133	532	1.33 (1.09–1.62)	2
Any antithrombotic	Female	Severe infection like	125	545	1.21 (0.98–1.48)	0
Any antithrombotic	Female	Infection any	271	1261	1.14 (0.99–1.33)	0
Any antithrombotic	Female	Hypertension	58	265	1.14 (0.85–1.52)	0
Anticoagulant	Male	Atrial arrhythmia	212	661	3.97 (3.35–4.70)	3
Anticoagulant	Male	Bleeding any	364	1584	3.14 (2.74–3.60)	3
Anticoagulant	Male	Major bleeding like	71	289	2.72 (2.08–3.55)	3
Anticoagulant	Male	Severe infection like	172	1371	1.39 (1.17–1.65)	2
Anticoagulant	Male	Infection any	264	2411	1.21 (1.05–1.40)	2

Anticoagulant	Male	Diarrhoea	84	717	1.26 (1.00–1.60)	1
Anticoagulant	Male	Hypertension	47	380	1.33 (0.97–1.81)	0
Anticoagulant	Male	Cytopenia	80	821	1.04 (0.82–1.32)	0
Anticoagulant	Female	Atrial arrhythmia	90	301	4.55 (3.53–5.86)	3
Anticoagulant	Female	Major bleeding like	36	137	3.66 (2.51–5.34)	3
Anticoagulant	Female	Bleeding any	162	967	2.73 (2.25–3.32)	3
Anticoagulant	Female	Cytopenia	53	447	1.63 (1.21–2.20)	2
Anticoagulant	Female	Infection any	125	1407	1.23 (1.00–1.51)	1
Anticoagulant	Female	Severe infection like	54	616	1.18 (0.88–1.58)	0
Anticoagulant	Female	Hypertension	23	300	1.02 (0.66–1.57)	0
Anticoagulant	Female	Diarrhoea	43	622	0.91 (0.66–1.25)	0
Antiplatelet	Male	Major bleeding like	77	283	2.06 (1.59–2.66)	3
Antiplatelet	Male	Bleeding any	328	1620	1.62 (1.42–1.85)	2
Antiplatelet	Male	Hypertension	75	352	1.60 (1.24–2.06)	2
Antiplatelet	Male	Atrial arrhythmia	136	737	1.39 (1.15–1.69)	2
Antiplatelet	Male	Severe infection like	227	1316	1.32 (1.13–1.53)	2
Antiplatelet	Male	Diarrhoea	118	683	1.29 (1.06–1.59)	2
Antiplatelet	Male	Infection any	331	2344	1.05 (0.92–1.20)	0
Antiplatelet	Male	Cytopenia	105	796	0.97 (0.78–1.20)	0
Antiplatelet	Female	Atrial arrhythmia	69	322	1.87 (1.43–2.45)	2
Antiplatelet	Female	Diarrhoea	99	566	1.53 (1.22–1.92)	2
Antiplatelet	Female	Bleeding any	157	972	1.44 (1.19–1.73)	2
Antiplatelet	Female	Major bleeding like	25	148	1.43 (0.93–2.20)	0
Antiplatelet	Female	Severe infection like	85	585	1.24 (0.98–1.58)	0
Antiplatelet	Female	Cytopenia	59	441	1.13 (0.85–1.50)	0
Antiplatelet	Female	Hypertension	38	285	1.13 (0.80–1.59)	0
Antiplatelet	Female	Infection any	177	1355	1.12 (0.94–1.34)	0

Supplementary Table S11. PT-level signal counts by exposure analysis.

Exposure analysis	PTs tested	Any positive algorithm	All three algorithms	ROR signal	PRR signal	IC signal
Anticoagulant	2197	192	152	181	158	192
Antiplatelet	2197	236	138	203	152	236
Any antithrombotic	2197	323	185	279	200	323
CYP3A inducer	2197	79	75	78	76	79
CYP3A inhibitor	2197	225	174	216	174	225
Moderate CYP3A inhibitor	2197	179	150	175	151	179
Any CYP3A modifier	2197	261	209	241	211	261
Strong CYP3A inhibitor	2197	90	87	89	88	90

Supplementary Table S12. Top 15 PT-level signals by exposure analysis.

Exposure analysis	Preferred term	a	c	ROR (95% CI)	Positive algorithms
Anticoagulant	INTERNATIONAL NORMALISED RATIO ABNORMAL	8	0	196.42 (11.33–3404.67)	3
Anticoagulant	POTENTIATING DRUG INTERACTION	3	0	80.64 (4.16–1561.81)	3
Anticoagulant	ANTICOAGULATION DRUG LEVEL ABOVE THERAPEUTIC	3	0	80.64 (4.16–1561.81)	3
Anticoagulant	PROTHROMBIN TIME ABNORMAL	3	0	80.64 (4.16–1561.81)	3
Anticoagulant	SOFT TISSUE SWELLING	3	0	80.64 (4.16–1561.81)	3
Anticoagulant	TEETH BRITTLE	3	0	80.64 (4.16–1561.81)	3
Anticoagulant	TONGUE BITING	4	1	46.12 (5.15–412.84)	3
Anticoagulant	RED BLOOD CELL TRANSFUSION	3	1	34.57 (3.59–332.50)	3
Anticoagulant	PROTHROMBIN TIME PROLONGED	3	1	34.57 (3.59–332.50)	3
Anticoagulant	AORTIC VALVE STENOSIS	3	1	34.57 (3.59–332.50)	3
Anticoagulant	FEBRILE INFECTION	3	1	34.57 (3.59–332.50)	3
Anticoagulant	DEFAECATION URGENCY	3	1	34.57 (3.59–332.50)	3
Anticoagulant	HAEMATOMA MUSCLE	3	1	34.57 (3.59–332.50)	3
Anticoagulant	INTERNATIONAL NORMALISED RATIO INCREASED	16	6	30.96 (12.10–79.22)	3
Anticoagulant	CYSTITIS ESCHERICHIA	4	2	23.06 (4.22–125.98)	3
Antiplatelet	AORTIC VALVE DISEASE	5	0	85.58 (4.73–1548.21)	3
Antiplatelet	LABELLED DRUG-DRUG INTERACTION MEDICATION ERROR	9	2	35.07 (7.57–162.42)	3
Antiplatelet	ANGINA UNSTABLE	6	2	23.35 (4.71–115.76)	3
Antiplatelet	GASTROINTESTINAL MUCOSAL DISORDER	3	1	23.32 (2.42–224.31)	3
Antiplatelet	EARLY SATIETY	3	1	23.32 (2.42–224.31)	3
Antiplatelet	INJECTION SITE HAEMATOMA	3	1	23.32 (2.42–224.31)	3
Antiplatelet	ENTEROCOLITIS HAEMORRHAGIC	3	1	23.32 (2.42–224.31)	3
Antiplatelet	CAROTID ARTERY STENOSIS	6	3	15.57 (3.89–62.28)	3
Antiplatelet	CYTOMEGALOVIRUS INFECTION REACTIVATION	5	3	12.97 (3.10–54.29)	3
Antiplatelet	HYPERKERATOSIS	6	4	11.67 (3.29–41.40)	3
Antiplatelet	DUODENAL ULCER HAEMORRHAGE	3	2	11.66 (1.95–69.82)	3
Antiplatelet	DYSLIPIDAEMIA	3	2	11.66 (1.95–69.82)	3
Antiplatelet	FAECES HARD	7	5	10.90 (3.46–34.37)	3
Antiplatelet	SMALL CELL LUNG CANCER	4	3	10.37 (2.32–46.36)	3
Antiplatelet	ARTERIOSCLEROSIS CORONARY ARTERY	4	3	10.37 (2.32–46.36)	3
Any antithrombotic	INTERNATIONAL NORMALISED RATIO ABNORMAL	8	0	79.06 (4.56–1370.16)	3
Any antithrombotic	AORTIC VALVE DISEASE	5	0	51.12 (2.83–924.67)	3

Any antithrombotic	RED BLOOD CELL TRANSFUSION	4	0	41.81 (2.25–776.80)	3
Any antithrombotic	INJECTION SITE HAEMATOMA	4	0	41.81 (2.25–776.80)	3
Any antithrombotic	BONE SWELLING	4	0	41.81 (2.25–776.80)	3
Any antithrombotic	POTENTIATING DRUG INTERACTION	3	0	32.51 (1.68–629.57)	3
Any antithrombotic	ANTICOAGULATION DRUG LEVEL ABOVE THERAPEUTIC	3	0	32.51 (1.68–629.57)	3
Any antithrombotic	HYPOCHROMIC ANAEMIA	3	0	32.51 (1.68–629.57)	3
Any antithrombotic	PROTHROMBIN TIME ABNORMAL	3	0	32.51 (1.68–629.57)	3
Any antithrombotic	SOFT TISSUE SWELLING	3	0	32.51 (1.68–629.57)	3
Any antithrombotic	TEETH BRITTLE	3	0	32.51 (1.68–629.57)	3
Any antithrombotic	CHRONIC LEFT VENTRICULAR FAILURE	3	0	32.51 (1.68–629.57)	3
Any antithrombotic	ORAL CONTUSION	5	1	23.24 (2.71–198.95)	3
Any antithrombotic	LABELLED DRUG-DRUG INTERACTION MEDICATION ERROR	9	2	20.93 (4.52–96.93)	3
Any antithrombotic	TONGUE BITING	4	1	18.58 (2.08–166.32)	3
CYP3A inducer	POLYMYOSITIS	17	1	996.20 (132.21–7506.59)	3
CYP3A inducer	TRICHOPHYTOSIS	5	0	622.42 (34.35–11277.73)	3
CYP3A inducer	TP53 GENE MUTATION	3	0	393.91 (20.31–7640.17)	3
CYP3A inducer	NATURAL KILLER-CELL LYMPHOBLASTIC LYMPHOMA	3	0	393.91 (20.31–7640.17)	3
CYP3A inducer	ANGIOCENTRIC LYMPHOMA	3	0	393.91 (20.31–7640.17)	3
CYP3A inducer	MYOSITIS	21	4	311.16 (106.25–911.28)	3
CYP3A inducer	BURKITT'S LYMPHOMA	3	1	169.04 (17.54–1628.98)	3
CYP3A inducer	TYROSINE KINASE MUTATION	3	1	169.04 (17.54–1628.98)	3
CYP3A inducer	TREATMENT FAILURE	30	12	152.07 (77.19–299.59)	3
CYP3A inducer	ENTEROCOCCAL INFECTION	4	2	113.00 (20.63–618.90)	3
CYP3A inducer	DERMATITIS EXFOLIATIVE GENERALISED	7	6	66.45 (22.22–198.72)	3
CYP3A inducer	DEVICE RELATED INFECTION	16	15	62.29 (30.55–126.98)	3
CYP3A inducer	LYMPHOPENIA	16	17	54.95 (27.54–109.65)	3
CYP3A inducer	WITHDRAWAL SYNDROME	4	6	37.66 (10.58–134.02)	3
CYP3A inducer	NECROTISING FASCIITIS	4	6	37.66 (10.58–134.02)	3
CYP3A inhibitor	MICROSPORIDIA INFECTION	18	2	168.60 (39.07–727.58)	3
CYP3A inhibitor	TRANSPLANT DYSFUNCTION	4	0	166.34 (8.95–3091.67)	3
CYP3A inhibitor	INHIBITORY DRUG INTERACTION	3	0	129.25 (6.67–2504.03)	3
CYP3A inhibitor	HYPOGEUSIA	3	0	129.25 (6.67–2504.03)	3
CYP3A inhibitor	HYPOSMIA	3	0	129.25 (6.67–2504.03)	3
CYP3A inhibitor	PROTHROMBIN TIME ABNORMAL	3	0	129.25 (6.67–2504.03)	3
CYP3A inhibitor	SOFT TISSUE SWELLING	3	0	129.25 (6.67–2504.03)	3
CYP3A inhibitor	SKIN TIGHTNESS	3	0	129.25 (6.67–2504.03)	3

CYP3A inhibitor	IODINE UPTAKE ABNORMAL	3	0	129.25 (6.67–2504.03)	3
CYP3A inhibitor	THYROTOXIC CRISIS	3	0	129.25 (6.67–2504.03)	3
CYP3A inhibitor	POLYMYOSITIS	15	3	93.40 (27.00–323.11)	3
CYP3A inhibitor	PEPTIC ULCER HAEMORRHAGE	5	1	92.53 (10.80–792.76)	3
CYP3A inhibitor	MYOSITIS	20	5	75.06 (28.12–200.39)	3
CYP3A inhibitor	DISSEMINATED MUCORMYCOSIS	7	2	64.89 (13.46–312.75)	3
CYP3A inhibitor	INTERVERTEBRAL DISCITIS	6	2	55.57 (11.20–275.65)	3
Moderate CYP3A inhibitor	HYPOGEUSIA	3	0	163.02 (8.41–3158.65)	3
Moderate CYP3A inhibitor	HYPOSMIA	3	0	163.02 (8.41–3158.65)	3
Moderate CYP3A inhibitor	PROTHROMBIN TIME ABNORMAL	3	0	163.02 (8.41–3158.65)	3
Moderate CYP3A inhibitor	SOFT TISSUE SWELLING	3	0	163.02 (8.41–3158.65)	3
Moderate CYP3A inhibitor	SKIN TIGHTNESS	3	0	163.02 (8.41–3158.65)	3
Moderate CYP3A inhibitor	IODINE UPTAKE ABNORMAL	3	0	163.02 (8.41–3158.65)	3
Moderate CYP3A inhibitor	THYROTOXIC CRISIS	3	0	163.02 (8.41–3158.65)	3
Moderate CYP3A inhibitor	DISSEMINATED MUCORMYCOSIS	7	2	81.93 (16.99–394.98)	3
Moderate CYP3A inhibitor	H1N1 INFLUENZA	6	2	70.14 (14.14–348.04)	3
Moderate CYP3A inhibitor	THYROXINE INCREASED	3	1	69.90 (7.26–672.72)	3
Moderate CYP3A inhibitor	COMA SCALE ABNORMAL	3	1	69.90 (7.26–672.72)	3
Moderate CYP3A inhibitor	CEREBRAL DISORDER	5	5	23.35 (6.75–80.81)	3
Moderate CYP3A inhibitor	ORAL MUCOSAL ERYTHEMA	3	3	23.30 (4.70–115.60)	3
Moderate CYP3A inhibitor	CHRONIC MYELOID LEUKAEMIA	3	3	23.30 (4.70–115.60)	3
Moderate CYP3A inhibitor	HAEMATOTOXICITY	8	9	20.82 (8.01–54.11)	3
Any CYP3A modifier	MICROSPORIDIA INFECTION	20	0	590.86 (35.72–9774.91)	3
Any CYP3A modifier	POLYMYOSITIS	17	1	244.52 (32.52–1838.80)	3
Any CYP3A modifier	TRICHOPHYTOSIS	5	0	156.79 (8.66–2837.01)	3
Any CYP3A modifier	TRANSPLANT DYSFUNCTION	4	0	128.19 (6.90–2382.21)	3
Any CYP3A modifier	HEPATIC HAEMATOMA	3	0	99.63 (5.14–1929.81)	3
Any CYP3A modifier	ADENOVIRUS INFECTION	3	0	99.63 (5.14–1929.81)	3
Any CYP3A modifier	INHIBITORY DRUG INTERACTION	3	0	99.63 (5.14–1929.81)	3
Any CYP3A modifier	HYPOGEUSIA	3	0	99.63 (5.14–1929.81)	3
Any CYP3A modifier	HYPOSMIA	3	0	99.63 (5.14–1929.81)	3
Any CYP3A modifier	PROTHROMBIN TIME ABNORMAL	3	0	99.63 (5.14–1929.81)	3
Any CYP3A modifier	SOFT TISSUE SWELLING	3	0	99.63 (5.14–1929.81)	3
Any CYP3A modifier	SKIN TIGHTNESS	3	0	99.63 (5.14–1929.81)	3
Any CYP3A modifier	IODINE UPTAKE ABNORMAL	3	0	99.63 (5.14–1929.81)	3
Any CYP3A modifier	THYROTOXIC CRISIS	3	0	99.63 (5.14–1929.81)	3

Any CYP3A modifier	B-LYMPHOCYTE COUNT DECREASED	3	0	99.63 (5.14–1929.81)	3
Strong CYP3A inhibitor	MICROSPORIDIA INFECTION	18	2	829.04 (191.23–3594.26)	3
Strong CYP3A inhibitor	TRANSPLANT DYSFUNCTION	4	0	778.74 (41.81–14505.08)	3
Strong CYP3A inhibitor	INHIBITORY DRUG INTERACTION	3	0	603.15 (31.07–11709.45)	3
Strong CYP3A inhibitor	POLYMYOSITIS	15	3	454.47 (130.67–1580.71)	3
Strong CYP3A inhibitor	PEPTIC ULCER HAEMORRHAGE	5	1	435.34 (50.66–3740.63)	3
Strong CYP3A inhibitor	MYOSITIS	20	5	371.73 (138.28–999.30)	3
Strong CYP3A inhibitor	H1N1 INFLUENZA	6	2	262.30 (52.67–1306.29)	3
Strong CYP3A inhibitor	DISSEMINATED MUCORMYCOSIS	6	3	174.86 (43.47–703.32)	3
Strong CYP3A inhibitor	INTERVERTEBRAL DISCITIS	5	3	145.10 (34.48–610.64)	3
Strong CYP3A inhibitor	DRUG LEVEL INCREASED	6	4	131.14 (36.77–467.73)	3
Strong CYP3A inhibitor	ACUTE GRAFT VERSUS HOST DISEASE	3	2	129.50 (21.54–778.57)	3
Strong CYP3A inhibitor	DEVICE RELATED INFECTION	15	16	85.16 (41.60–174.34)	3
Strong CYP3A inhibitor	TREATMENT FAILURE	20	22	84.41 (45.42–156.89)	3
Strong CYP3A inhibitor	LYMPHOPENIA	15	18	75.69 (37.68–152.06)	3
Strong CYP3A inhibitor	SPINAL CORD COMPRESSION	5	6	72.54 (21.99–239.33)	3

Supplementary Table S13. Exploratory univariable death and hospitalization reporting models.

Model	Variable	Term	OR (95% CI)	p value
Death reporting	cyp3a_modifier	cyp3a_modifierTRUE	1.38 (1.19–1.58)	7.78e-06
Death reporting	cyp3a_inhibitor	cyp3a_inhibitorTRUE	1.14 (0.97–1.34)	0.115
Death reporting	cyp3a_inducer	cyp3a_inducerTRUE	1.98 (1.56–2.51)	1.57e-08
Death reporting	antithrombotic	antithromboticTRUE	0.86 (0.78–0.96)	0.005
Death reporting	anticoagulant	anticoagulantTRUE	0.91 (0.79–1.05)	0.195
Death reporting	antiplatelet	antiplateletTRUE	0.85 (0.75–0.97)	0.013
Death reporting	AGE_GROUP	AGE_GROUP65-74	1.20 (1.08–1.33)	0.000946
Death reporting	AGE_GROUP	AGE_GROUP75+	1.79 (1.62–1.98)	1.34e-30
Death reporting	SEX_STD	SEX_STDMale	1.34 (1.24–1.46)	1.34e-12
Death reporting	SEX_STD	SEX_STDUnknown	2.27 (1.60–3.17)	2.14e-06
Death reporting	diagnosis_group	diagnosis_groupCLL+SLL	1.01 (0.65–1.50)	0.97
Death reporting	diagnosis_group	diagnosis_groupSLL	1.33 (1.10–1.61)	0.00335
Hospitalization reporting	cyp3a_modifier	cyp3a_modifierTRUE	1.55 (1.38–1.73)	9.83e-15
Hospitalization reporting	cyp3a_inhibitor	cyp3a_inhibitorTRUE	1.58 (1.39–1.78)	5.98e-13
Hospitalization reporting	cyp3a_inducer	cyp3a_inducerTRUE	1.51 (1.22–1.86)	0.000111

Hospitalization reporting	antithrombotic	antithromboticTRUE	1.64 (1.53–1.76)	4.56e-42
Hospitalization reporting	anticoagulant	anticoagulantTRUE	1.80 (1.63–2.00)	7.73e-30
Hospitalization reporting	antiplatelet	antiplateletTRUE	1.52 (1.39–1.66)	1.2e-21
Hospitalization reporting	AGE_GROUP	AGE_GROUP65-74	1.13 (1.06–1.21)	0.000513
Hospitalization reporting	AGE_GROUP	AGE_GROUP75+	1.11 (1.04–1.19)	0.00327
Hospitalization reporting	SEX_STD	SEX_STDMale	1.18 (1.12–1.25)	7.6e-09
Hospitalization reporting	SEX_STD	SEX_STDUnknown	0.33 (0.23–0.47)	1.46e-09
Hospitalization reporting	diagnosis_group	diagnosis_groupCLL+SLL	1.26 (0.94–1.71)	0.124
Hospitalization reporting	diagnosis_group	diagnosis_groupSLL	0.62 (0.53–0.72)	2.08e-09

Supplementary Table S14. Report-level exposure co-occurrence matrix. Cells show the number of unique reports positive for both the row exposure and the column exposure. Diagonal cells show total exposed reports for each exposure indicator.

**Exposure**	**CYP3A modifier**	**CYP3A inhibitor**	**Strong CYP3A inhibitor**	**Moderate CYP3A inhibitor**	**CYP3A inducer**	**Antithrombotic**	**Anticoagulant**	**Antiplatelet**
CYP3A modifier	1373	1075	242	862	367	528	318	281
CYP3A inhibitor	1075	1075	242	862	69	455	282	237
Strong CYP3A inhibitor	242	242	242	29	30	58	30	32
Moderate CYP3A inhibitor	862	862	29	862	42	408	259	212
CYP3A inducer	367	69	30	42	367	89	47	52
Antithrombotic	528	455	58	408	89	3701	1670	2382
Anticoagulant	318	282	30	259	47	1670	1670	351
Antiplatelet	281	237	32	212	52	2382	351	2382

Supplementary Table S15. Exploratory joint logistic models for death and hospitalization reporting. Models include CYP3A modifier co-exposure, antithrombotic co-exposure, age group, sex and diagnosis group. Reference categories are no co-exposure, age 18-64 years, female sex and CLL diagnosis group. Results are descriptive and should not be interpreted as causal estimates.

**Model**	**Term**	**OR (95% CI)**	**p value**
Death reporting	CYP3A modifier	1.48 (1.28-1.71)	9.11e-08
Death reporting	Antithrombotic	0.74 (0.67-0.83)	5.59e-08
Death reporting	Age 65-74 vs 18-64	1.23 (1.11-1.37)	0.000133
Death reporting	Age >=75 vs 18-64	1.92 (1.74-2.13)	8.52e-37

Death reporting	Male vs female	1.41 (1.29-1.53)	5.23e-16
Death reporting	Unknown sex vs female	2.32 (1.65-3.27)	1.44e-06
Death reporting	CLL+SLL vs CLL	1.04 (0.68-1.58)	0.857
Death reporting	SLL vs CLL	1.31 (1.08-1.59)	0.00636
Hospitalization reporting	CYP3A modifier	1.40 (1.25-1.57)	4.68e-09
Hospitalization reporting	Antithrombotic	1.57 (1.46-1.69)	2.78e-33
Hospitalization reporting	Age 65-74 vs 18-64	1.09 (1.02-1.17)	0.0137
Hospitalization reporting	Age >=75 vs 18-64	1.06 (0.99-1.14)	0.0952
Hospitalization reporting	Male vs female	1.17 (1.10-1.24)	8.60e-08
Hospitalization reporting	Unknown sex vs female	0.33 (0.23-0.48)	2.71e-09
Hospitalization reporting	CLL+SLL vs CLL	1.23 (0.91-1.66)	0.185
Hospitalization reporting	SLL vs CLL	0.60 (0.52-0.71)	4.98e-10
